# Highly regio- and stereoselective phosphinylphosphination of terminal alkynes with tetraphenyldiphosphine monoxide under radical conditions

**DOI:** 10.3762/bjoc.17.72

**Published:** 2021-04-20

**Authors:** Dat Phuc Tran, Yuki Sato, Yuki Yamamoto, Shin-ichi Kawaguchi, Shintaro Kodama, Akihiro Nomoto, Akiya Ogawa

**Affiliations:** 1 Department of Applied Chemistry, Graduate School of Engineering, Osaka Prefecture University, 1-1 Gakuen-cho, Nakaku, Sakai, Osaka 599-8531, Japan; 2 Center for Education and Research in Agricultural Innovation, Faculty of Agriculture, Saga University, 152-1 Shonan-cho, Karatsu, Saga 847-0021, Japan

**Keywords:** (*E*)-1,2-bis(diphenylphosphino)ethylene derivative, radical addition, stereoselective phosphinylphosphination, terminal alkyne, tetraphenyldiphosphine monoxide

## Abstract

The homolytic cleavage of the P^V^(O)–P^III^ bond in tetraphenyldiphosphine monoxide simultaneously provides both pentavalent and trivalent phosphorus-centered radicals with different reactivities. The method using V-40 as an initiator is successfully investigated for the regio- and stereoselective phosphinylphosphination of terminal alkynes giving the corresponding *trans*-isomers of 1-diphenylphosphinyl-2-diphenylthiophosphinyl-1-alkenes in good yields. The protocol can be applied to a wide variety of terminal alkynes including both alkyl- and arylalkynes.

## Introduction

**Scheme 1:**
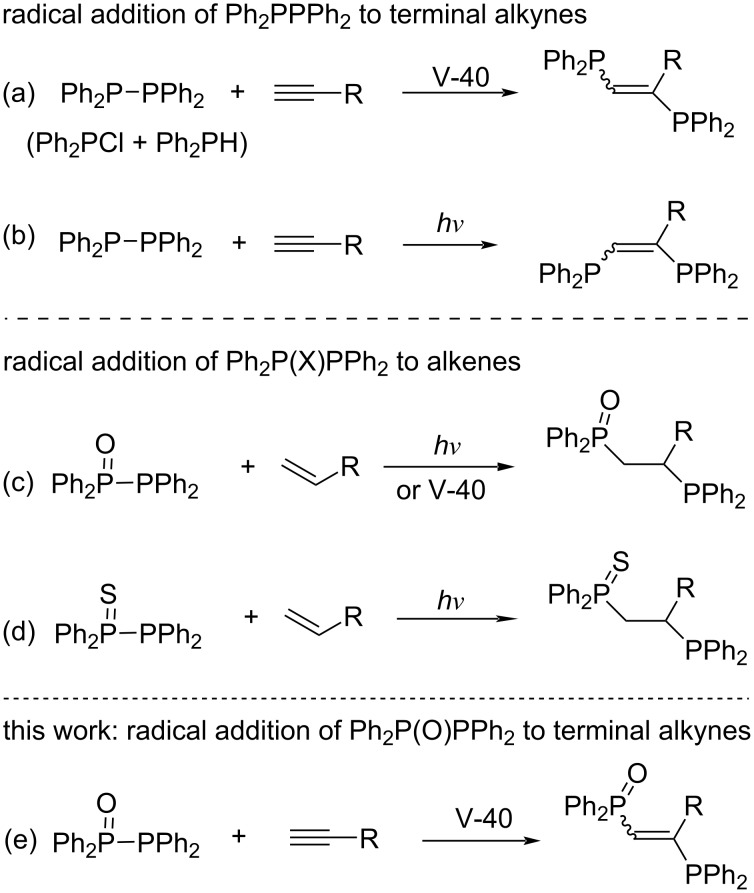
Radical addition of Ph_2_PPPh_2_ and Ph_2_P(X)PPh_2_ to unsaturated C–C bonds.

Organophosphorus compounds are an essential class of compounds in catalytic technologies, biochemistry, and materials [1–6]. In particular, in coordination chemistry and catalyst chemistry, organophosphorus compounds such as triorganylphosphines are widely used as typical monodentate ligands for many metals [7–8]. In addition, diphosphines such as 1,2-bis(diphenylphosphino)ethane (Ph_2_PCH_2_CH_2_PPh_2_, dppe) are employed as bidentate ligands and are effective in controlling important catalytic reactions such as cross-coupling reactions [9–18]. 1,2-Bis(diphenylphosphino)ethylene (Ph_2_PCH=CHPPh_2_, dppen) is among bidentate diphosphine ligands having a rigid structure and has increased attention as a useful and effective ligand in coordinating with various metals [19–20]. (*Z*)-Ph_2_PCH=CHPPh_2_ is very important as a bidentate ligand for many mononuclear complexes [21–23]. On the other hand, (*E*)-Ph_2_PCH=CHPPh_2_ acts as a monodentate ligand for mononuclear complexes, but it is highly attractive because a hierarchical structure can be constructed by cross-linking between two metals [24–26]. Considering the characteristics of the coordination form between the (*E*)- and (*Z*)-isomers, the development of a synthetic method for the highly selective synthesis of the (*E*)- or (*Z*)-isomers is strongly desired [27–30]. Furthermore, since it was reported that the introduction of a substituent into the ethylene moiety has a great effect on the catalytic activity [31], it is considered important to synthesize derivatives having various substituents on the ethylene moiety. In particular, synthetic methods that do not use metal catalysts and reagents are expected to be very effective in manufacturing precision materials and pharmaceuticals [32–37]. To develop metal-free methods for the synthesis of 1,2-bis(diphenylphosphino)ethylenes, we have recently been conducting systematic research on the radical addition of P–P bond compounds to unsaturated carbon–carbon bonds [38–44]. Early studies have found that the radical addition of tetraphenyldiphosphine (Ph_2_PPPh_2_) to alkynes using a radical initiator such as 1,1'-azobis(cyclohexane-1-carbonitrile) (V-40) (Scheme 1a) [45] or upon photoirradiation (Scheme 1b) [38] yields *vic*-bis(diphenylphosphino)alkenes in good yields. Unfortunately, this photoinduced reaction of Ph_2_PPPh_2_ was not applicable to alkenes [42]. To change the reactivity of the P–P bond, therefore, when the combination of pentavalent phosphorus and trivalent phosphorus was examined, it was found that the desired radical addition of Ph_2_P(X)PPh_2_ (X = O, S) to alkenes successfully occurred [42–43] (Scheme 1c and 1d).

However, in the case of Ph_2_P(O)PPh_2_, its absorption is located at a shorter wavelength (λ_max_ = 318 nm) and the absorption intensity is lower than those of Ph_2_PPPh_2_ and Ph_2_P(S)PPh_2_ [46]. Indeed, the photoinduced addition of Ph_2_P(O)PPh_2_ to alkynes required prolonged reaction times (>40 h), and the scope of this alkyne addition was unexamined. Thus, we examined in detail the radical addition of Ph_2_P(O)PPh_2_ to alkynes and found that the desired radical addition proceeds efficiently using a radical initiator instead of light irradiation, providing 1-(diphenylphosphinyl)-2-(diphenylphosphino)-1-alkenes (Scheme 1e).

## Results and Discussion

**Scheme 2:**
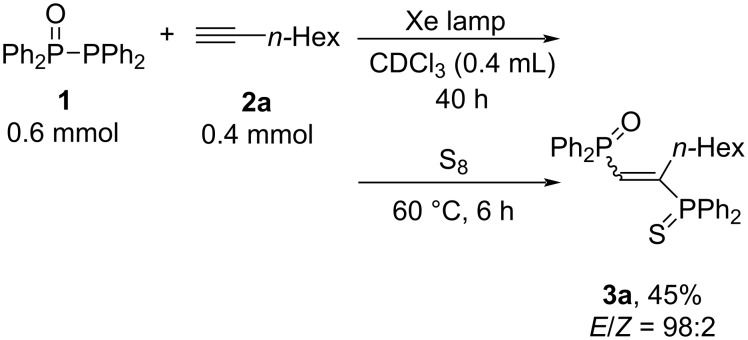
The addition of Ph_2_P(O)PPh_2_ (**1**) to 1-octyne (**2a**).

First, a mixture of Ph_2_P(O)PPh_2_ (**1**, 0.6 mmol) and 1-octyne (**2a**, 0.4 mmol) was irradiated with a xenon lamp. After 40 hours, sulfurization of the addition product was performed to afford the phosphinylphosphination product **3a** in 45% yield, confirmed by ^31^P NMR spectroscopy (Scheme 2) [47].

Next, the reaction was carried out varying the reaction parameters such as the light source and the ratio of the radical initiator (V-40)/**2a** (Table 1). Using a xenon lamp as an artificial solar source [48–49], **3a** was produced in 45% yield (Table 1, entry 1). Using UV light irradiation with a high-pressure mercury lamp [50], **3a** was produced in 28% yield (Table 1, entry 2). However, **3a** was obtained only in trace amounts upon irradiation with a tungsten lamp [51] (Table 1, entry 3). These results indicate that Ph_2_P• [52–55] and/or Ph_2_P(O)• [56–59] radicals were generated by the irradiation with light in the near-UV region. When benzene was used instead of CDCl_3_ under xenon lamp irradiation, the reaction did not proceed because **1** was less soluble in benzene than CDCl_3_ (Table 1, entry 4). The radical initiator, V-40, was found to be an appropriate initiator for the generation of phosphorus-centered radicals [38,42]. The ratio of V-40/**2a** was important to depress the formation of self-polymerization of **2a** (Table 1, entries 5–7). The results showed that the best amount of V-40 toward **2a** was 10 mol %. Besides, the side product **3a’** is found up to 8% yield under photoirradiation. Considering that in our previously reported radical addition reactions of Ph_2_P(S)PPh_2_ and Ph_2_P(S)P(S)Ph_2_ to alkynes [44], the *E*/*Z* ratios were about 9:1 and 8:2, respectively, it should be noted that the present addition of Ph_2_P(O)PPh_2_ **1** to alkynes afforded (*E*)-isomers with an excellent stereoselectivity (greater than 95:5) [60].

**Table 1:** Phosphinylphosphination of terminal alkyne **2a** with **1** under different reaction parameters.

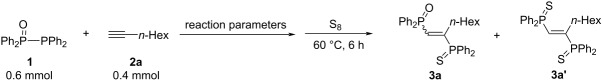			

entry	reaction parameters	yield of **3a**^a^ (%), *E*/*Z*	yield of **3a’**^a^ (%)

1	xenon lamp, CDCl_3_ (0.4 mL), 40 h	45, 98:2	8
2	high-pressure mercury lamp, CDCl_3_ (0.4 mL), 40 h	28, 97:3	4
3	tungsten lamp, CDCl_3_ (0.4 mL), 40 h	trace	–
4	xenon lamp, benzene (0.4 mL), 40 h	trace	–
5	V-40 (5 mol %), benzene (0.6 mL), 80 °C, 22 h	40, 99:1	2
6	V-40 (10 mol %), benzene (0.6 mL), 80 °C, 22 h	73 (67), 99:1	3
7	V-40 (15 mol %), benzene (0.6 mL), 80 °C, 22 h	64, 100:0	5

^a^Determined by ^31^P NMR spectroscopy. V-40 = 1,1’-azobis(cyclohexane-1-carbonitrile). Isolated yield is shown in parentheses.

**Scheme 3:**
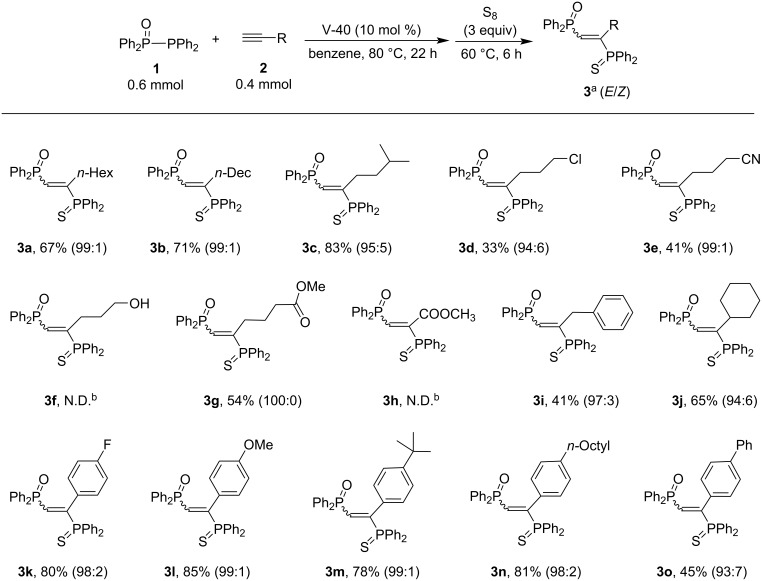
Phosphinylphosphination of various terminal alkynes **2** with **1**. ^a^Isolated yields. V-40 = 1,1’-azobis(cyclohexane-1-carbonitrile). ^b^Repeated 2 times.

The phosphinylphosphination of various terminal alkynes **2** with Ph_2_P(O)PPh_2_ **1** was conducted under the optimization conditions (Scheme 3). Terminal alkylalkynes **2a**, **2b**, and **2c** reacted efficiently with **1** to give the corresponding adducts **3a**, **3b**, and **3c** in moderate to good yields (67%, 71%, and 83%, respectively) with excellent stereoselectivity (*E*/*Z* = 95:5–99:1). Terminal alkylalkynes with chloro (**2d**), cyano (**2e**), and ester (**2g**) groups provided the corresponding adducts **3d**, **3e**, and **3g** in 33%, 41%, and 54% yields, respectively (*E*/*Z* = 94:6–100:0). In sharp contrast, the presence of a hydroxy group inhibited the desired phosphinylphosphination (see, alkyne **2f**), probably because of the decomposition of Ph_2_P(O)PPh_2_. Furthermore, an electron-deficient alkyne such as methyl propiolate (**2h**) failed to provide the desired adduct (**3h**) [61]. 3-Phenyl-1-propyne (**2i**) and cyclohexylacetylene (**2j**) gave **3i** and **3j** in 41% and 65% yields, respectively. Again, an excellent stereoselectivity was observed. Moreover, terminal arylalkynes **2k–o** were also tolerated under the conditions to afford the desired adducts **3k–o** in moderate to good yields with high stereoselectivity.

**Scheme 4:**
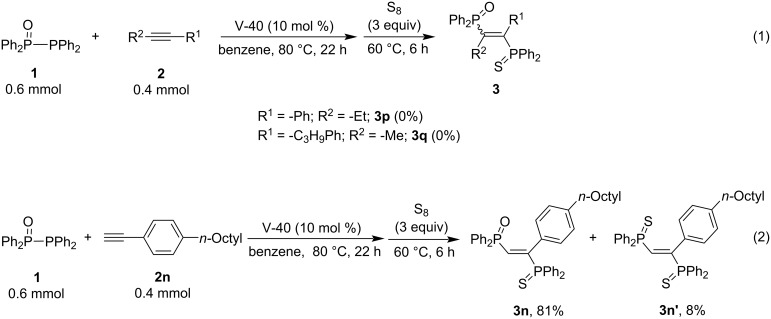
Attempted radical addition to internal alkynes and insight into the addition to **2n**.

We also investigated the phosphinylphosphination of some internal alkynes, **2p** and **2q,** with Ph_2_P(O)PPh_2_, but did not afford any adduct (the starting alkynes were recovered unchanged) (Scheme 4). This is most probably because the internal alkynes are sterically bulkier than terminal alkynes, and therefore, the addition did not proceed (Scheme 4, reaction 1). On the other hand, reaction 2 in Scheme 4 indicates an example of the phosphinylphosphination of a terminal alkyne. The detailed analysis of the products in this reaction revealed the formation of 8% of the addition product **3n’**, which might be formed by the addition of Ph_2_P• to the alkyne. Noteworthy is that the capture of carbon radicals occurred only at the trivalent phosphorus site of Ph_2_P(O)PPh_2_. Therefore, the initiation step might also proceed via the attack of the carbon radical generated from V-40 at the trivalent phosphorus site to form Ph_2_P(O)• selectively.

**Scheme 5:**
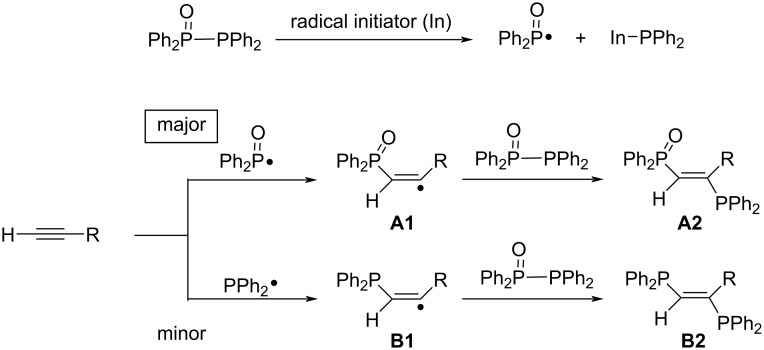
A plausible reaction pathway for the radical addition of Ph_2_P(O)PPh_2_ to terminal alkynes.

With this information in mind, a plausible reaction pathway is shown in Scheme 5. Decomposition of the radical initiator (V-40) generates In•, which attacks selectively at the trivalent phosphorus atom of Ph_2_P(O)PPh_2_ to form Ph_2_P(O)•. Then, Ph_2_P(O)• adds to the terminal carbon of an alkyne to afford the carbon-centered radical **A1**. Radical **A1** is captured by Ph_2_P(O)PPh_2_ to provide **A2**, regio- and stereoselectively [62].

## Conclusion

In conclusion, a highly regio- and stereoselective phosphinylphosphination of alkynes with Ph_2_P(O)PPh_2_ has been successfully developed. The radical initiator V-40 can be used to selectively generate Ph_2_P(O)• as an important species to regioselectively afford 1-phosphinyl-2-phosphinoalkenes. This method can be applied to a wide range of terminal alkynes. We believe that the excellent stereoselectivity to give (*E*)-isomers is effective for the stereoselective synthesis of (*E*)-bis(diphenylphosphino)ethylenes.

## Experimental

### General comments

Unless otherwise state, materials were obtained from commercial suppliers and purified by distillation. ^1^H NMR spectra were recorded on a JEOL JNM-ECS400 (400 MHz) spectrometer or JEOL JNM-ECX400 (400 MHz) FT spectrometer in CDCl_3_ as the solvent with tetramethylsilane (TMS) as an internal standard. ^13^C NMR spectra were taken mainly on JEOL JNM-ECS400 (100 MHz) and JEOL JNM-ECX400 (100 MHz) FT spectrometers in CDCl_3._^31^P NMR spectra were recorded on a JEOL JNM-ECX400 (162 MHz) FT spectrometer in CDCl_3_ with 85% H_3_PO_4_ solution as an external standard or a Bruker BioSpin Ascend 400 spectrometer (162 MHz). ^19^F NMR spectra were recorded on a Bruker BioSpin Ascend 400 spectrometer (377 MHz). IR spectra were recorded on JASCO FT/IR-680Plus instrument. High-resolution mass spectra (HRMS) were recorded on a Bruker micrOTOF II ESI(+)/TOF instrument.

### General procedure for the phosphinylphosphination of alkynes

Ph_2_P(O)PPh_2_ (**1**, 0.6 mmol) and an alkyne (**2**, 0.4 mmol) were placed in a Schlenk tube with CDCl_3_ or benzene (super dehydrated) under argon atmosphere. V-40 was added to the mixture, and then the reaction was heated at 80 °C and stirred for 22 h. After the reaction was complete, sulfur (3 equiv) was added under inert atmosphere and then the mixture was stirred at 60 °C for 6 h to provide the stable adduct **3**. The purification of the products was performed by silica gel column chromatography using isohexane/MeOAc as an eluent.

## Supporting Information

File 1 Characterization data and copies of NMR spectra.
