# Supplementary material for: Highly regio- and stereoselective phosphinylphosphination of terminal alkynes with tetraphenyldiphosphine monoxide under radical conditions
Source: Beilstein J Org Chem. 2021 Apr 20;17:866–72. doi: 10.3762/bjoc.17.72 (PMC8077611; doi:10.3762/bjoc.17.72)

BEILSTEIN JOURNAL OF ORGANIC CHEMISTRY

Supporting Information

for

Highly regio- and stereoselective phosphinylphosphination of terminal alkynes with tetraphenyldiphosphine monoxide under radical conditions

Dat Phuc Tran, Yuki Sato, Yuki Yamamoto, Shin-ichi Kawaguchi, Shintaro Kodama, Akihiro Nomoto and Akiya Ogawa

Beilstein J. Org. Chem. **2021,** *17,* 866–872. doi:10.3762/bjoc.17.72

Characterization data and copies of NMR spectra

License and Terms: This is a supporting information file under the terms of the Creative Commons Attribution License (https://creativecommons.org/licenses/by/4.0). Please note that the reuse, redistribution and reproduction in particular requires that the author(s) and source are credited and that individual graphics may be subject to special legal provisions.
The license is subject to the *Beilstein Journal of Organic Chemistry* terms and conditions: (https://www.beilstein-journals.org/bjoc/terms)

CONTENTS

1. Characterization data S3−S10

2. Copies of NMR spectra S11−S50

1. Characterization Data

(*E*)- 1-(diphenylphosphinyl)-2-(diphenylthiophosphinyl)-oct-1-ene (3a)

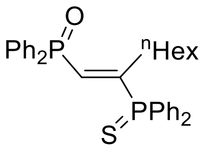

Colorless oil; ^{1}H NMR (400 MHz, CDCl$_3$): δ 7.78-7.72 (m, 4H), 7.70-7.65 (m, 4H), 7.54-7.48 (m, 4H), 7.46-7.41 (m, 8H), 7.19 (dd, J_{H-P} = 27.9, 24.3 Hz, 1H), 2.90-2.82 (m, 2H), 1.09-0.86 (m, 8H), 0.72 (t, J = 7.3 Hz, 3H); ^{13}C NMR (100 MHz, CDCl$_3$): δ 158.1 (d, J_{C-P} = 58.5 Hz), 135.1 (dd, J_{C-P} = 88.2, 7.7 Hz), 133.7 (d, J = 105.4 Hz), 132.3 (d, J = 10.5 Hz), 131.5 (d, J = 115.0 Hz), 131.7, 130.9 (d, J = 9.6 Hz), 128.84 (dd, J = 12.5, 8.6 Hz), 31.3 (dd, J = 10.0, 5.8 Hz), 31.2, 31.1, 29.5, 22.4, 14.03; ^{31}P NMR (162 MHz, CDCl$_3$): δ 49.6 (d, J_{P-P} = 56.4 Hz), 20.26 (d, J_{P-P} = 56.4 Hz); IR (KBr, cm^{-1}): 3055, 2954, 2928, 2855, 1437,1201, 1117, 1102, 744, 719, 693, 640; HRMS (ESI+) *m/z* calcd for $C_{32}H_{34}NaOP_2S$ [M+Na]$^+$: 551.1703, found: 551.1696.

(*E*)- 1-(diphenylphosphinyl)-2-(diphenylthiophosphinyl)dodec-1-ene (3b)

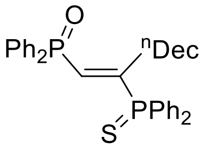

Colorless oil; ^{1}H NMR (400 MHz, CDCl$_3$): δ 7.79-7.71 (m, 4H), 7.70-7.65 (m, 4H), 7.54-7.48 (m, 4H), 7.46-7.41 (m, 8H), 7.20 (dd, J_{H-P} = 27.9, 24.0 Hz, 1H), 2.90-2.81 (m, 2H), 1.33-0.90 (m, 16H), 0.86 (t, J = 7.3 Hz, 3H); ^{13}C NMR (100 MHz, CDCl$_3$): δ 158.1 (d, J_{C-P} = 58.5 Hz), 135.1 (dd, J_{C-P} = 88.2, 7.6 Hz), 133.7 (d, J = 104.9 Hz), 132.3 (d, J = 10.5 Hz), 131.5 (d, J = 115.4 Hz), 131.7, 130.9 (d, J = 9.5 Hz), 128.8 (dd, J = 11.4, 8.6 Hz), 32.0, 31.3 (dd, J = 9.5, 6.7 Hz), 31.1, 29.9, 29.6, 29.44, 29.36, 29.0, 22.8, 14.2; ^{31}P NMR (162 MHz, CDCl$_3$): δ 48.9 (d, J_{P-P} = 58.6 Hz), 19.6 (d, J_{P-P} = 58.6 Hz); IR (KBr, cm^{-1}): 3054, 2924, 2852, 1436, 1202,

1117, 1102, 744, 719, 693, 640, 545, 527, 498; HRMS (ESI+) *m/z* calcd for $C_{36}H_{42}NaOP_2S$ $[M+Na]^+$: 607.2329, found: 607.2328.

(*E*)- 1-(diphenylphosphinyl)-2-(diphenylthiophosphinyl)-5-methylhex-1-ene (3c)

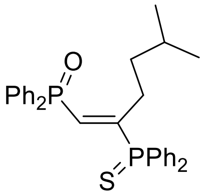

Colorless oil; ^{1}H NMR (400 MHz, $CDCl_3$): δ 7.77-7.72 (m, 4H), 7.70-7.66 (m, 4H), 7.54-7.49 (m, 4H), 7.47-7.42 (m, 8H), 7.27 (dd, $J_{H\text{-}P}$ = 27.9, 23.8 Hz, 1H), 2.90-2.81 (m, 2H), 1.28-1.17 (m, 1H), 0.94-0.87 (m, 2H), 0.56 (d, *J* = 6.9 Hz, 6H); ^{13}C NMR (100 MHz, $CDCl_3$): δ 158.3 (d, $J_{C\text{-}P}$ = 57.5 Hz), 135.3 (dd, $J_{C\text{-}P}$ = 88.2, 7.7 Hz), 133.8 (d, *J* = 105.4 Hz), 132.4 (d, *J* = 9.6 Hz), 131.5 (d, *J* = 115.0 Hz), 131.7, 130.9 (d, *J* = 10.5 Hz), 128.8 (dd, *J* = 12.5, 6.7 Hz), 39.6, 29.5 (dd, *J* = 9.6, 7.7 Hz), 28.6, 22.0; ^{31}P NMR (162 MHz, $CDCl_3$): δ 49.6 (d, $J_{P\text{-}P}$ = 56.4 Hz), 20.0 (d, $J_{P\text{-}P}$ = 56.4 Hz); IR (KBr, cm^{-1}): 2955, 1436, 1198, 1102, 745, 719, 693; HRMS (ESI+) *m/z* calcd for $C_{31}H_{32}NaOP_2S$ $[M+Na]^+$: 537.1547, found: 537.1547.

(*E*)-1-(diphenylphosphinyl)-2-(diphenylthiophosphinyl)-5-chloropent-1-ene (3d)

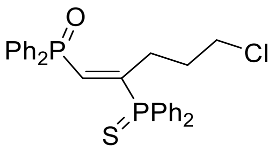

Colorless oil; ^{1}H NMR (400 MHz, $CDCl_3$): δ 7.77-7.71 (m, 4H), 7.69-7.34 (m, 4H), 7.56-7.52 (m, 4H), 7.48-7.44 (m, 8H), 7.27 (dd, $J_{H\text{-}P}$ = 28.0, 24.0 Hz, 1H), 3.28 (t, *J* = 6.3 Hz, 2H), 3.03-2.94 (m, 2H), 1.63-1.56 (m, 2H); ^{13}C NMR (100 MHz, $CDCl_3$): δ 156.7 (d, $J_{C\text{-}P}$ = 59.1 Hz), 136.0 (dd, $J_{C\text{-}P}$ = 87.3, 6.7 Hz), 133.1 (d, *J* = 104.9 Hz), 132.3 (d, *J* = 9.5 Hz), 130.9 (d, *J* = 9.5 Hz), 130.7 (d, *J* = 83.9 Hz), 129.0 (dd, *J* = 12.4, 3.8 Hz), 44.73, 33.7, 28.9 (dd, *J* = 10.0, 6.7 Hz); ^{31}P NMR (162 MHz, $CDCl_3$): δ 48.9 (d, $J_{P\text{-}P}$ = 54.1 Hz), 20.4 (d, $J_{P\text{-}P}$ = 54.1 Hz); IR (KBr,

cm^{-1}): 3054, 2925, 1436, 1198, 1101, 720, 693, 640, 527, 544; HRMS (ESI+) *m/z* calcd for $C_{29}H_{27}ClNaOP_2S$ [M+Na]$^+$: 543.0844, found: 543.0843.

(*E*)-1-(diphenylphosphinyl)-2-(diphenylthiophosphinyl)-5-cyanopent-1-ene (3e)

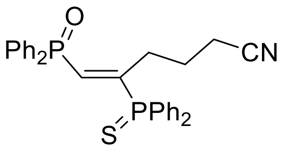

Colorless oil; ^{1}H NMR (400 MHz, $CDCl_3$): δ 7.77-7.70 (m, 4H), 7.67-7.62 (4H, m), 7.58-7.52 (m, 4H), 7.50-7.44 (m, 8H), 7.22 (dd, $J_{H\text{-}P}$ = 27.1, 24.0 Hz, 1H), 3.02-2.94 (m, 2H), 2.18 (t, *J* = 7.2 Hz, 2H), 1.55-1.47 (m, 2H); ^{13}C NMR (100 MHz, $CDCl_3$): δ 155.8 (d, $J_{C\text{-}P}$ = 60.1 Hz), 136.3 (dd, $J_{C\text{-}P}$ = 86.3, 7.6 Hz), 133.1 (d, *J* = 105.9 Hz), 132.4 (d, *J* = 2.9 Hz), 132.3 (d, *J* = 10.5 Hz), 130.9 (d, *J* = 9.5 Hz), 130.6 (d, *J* = 83.9 Hz), 129.1 (d, *J* = 12.4 Hz), 119.2, 29.9 (dd, *J* = 10.0, 6.7 Hz), 27.0, 17.2; ^{31}P NMR (162 MHz, $CDCl_3$): δ 48.6 (d, $J_{P\text{-}P}$ = 55.2 Hz), 20.0 (d, $J_{P\text{-}P}$ = 55.2 Hz); IR (KBr, cm^{-1}): 3054, 2937, 2245, 1436, 1197, 1102, 745, 720, 693, 642; HRMS (ESI+) *m/z* calcd for $C_{30}H_{27}NNaOP_2S$ [M+Na]$^+$: 534.1186, found: 534.1186.

(*E*)-1-(diphenylphosphinyl)-2-(diphenylthiophosphinyl)-methylhex-1-enoate (3g)

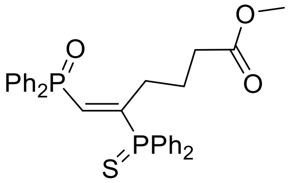

Colorless oil; ^{1}H NMR (400 MHz, $CDCl_3$): δ 7.76-7.71 (m, 4H), 7.67-7.62 (m, 4H), 7.53-7.49 (m, 4H), 7.47-7.42 (m, 8H), 7.14 (dd, $J_{H\text{-}P}$ = 27.6, 24.0 Hz, 1H), 3.52 (s, 3H), 2.97-2.88 (m, 2H), 2.09 (t, *J* = 7.2 Hz, 2H), 1.51-1.43 (m, 2H); ^{13}C NMR (100 MHz, $CDCl_3$): δ 173.2, 157.1 (d, $J_{C\text{-}P}$ = 58.2 Hz), 135.7 (dd, $J_{C\text{-}P}$ = 87.7, 6.7 Hz), 133.3 (d, *J* = 104.9 Hz), 132.3, 132.2 (d, *J* = 4.8 Hz), 130.87 (d, *J* = 83.9 Hz), 130.88 (d, *J* = 9.5 Hz), 128.9 (dd, *J* = 12.4, 7.6 Hz), 51.4, 34.1, 30.2 (dd, *J* = 9.5, 6.7 Hz), 26.5; ^{31}P NMR (162 MHz, $CDCl_3$): δ 48.9 (d, $J_{P\text{-}P}$ = 58.2 Hz),

20.0 (d, J_{P-P} = 58.2 Hz); IR (KBr, cm^{-1}): 3054, 2937, 1732, 1436, 1196, 1101, 744, 719, 693; HRMS (ESI+) *m/z* calcd for $C_{31}H_{30}NaO_3P_2S$ $[M+Na]^+$: 567.1289, found: 567.1289.

(*E*)-1-(diphenylphosphinyl)-2-(diphenylthiophosphinyl)-3-phenylprop-1-ene (3i)

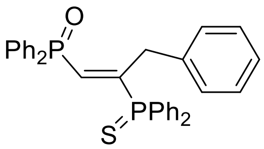

White solide; mp. 124-125 °C; ^1H NMR (400 MHz, $CDCl_3$): δ 7.63-7.56 (m, 8H), 7.52-7.48 (m, 2H), 7.44-7.38 (m, 6H), 7.31-7.27 (m, 4H), 7.04 (dd, J_{H-P} = 25.8, 23.1 Hz, 1H), 6.99 (d, *J* = 5.9 Hz, 2H), 6.87-6.80 (m, 3H), 4.89 (d, *J* = 19.0 Hz, 2H); ^{13}C NMR (100 MHz, $CDCl_3$): δ 156.4 (d, J_{C-P} = 59.1 Hz), 136.6, 136.0 (dd, J_{C-P} = 87.7, 6.7 Hz), 133.3 (d, *J* = 104.9 Hz), 132.2 (d, *J* = 10.5 Hz), 132.1 (d, *J* = 2.9 Hz), 131.8 (d, *J* = 2.9 Hz), 130.9 (d, *J* = 9.5 Hz), 130.7 (d, *J* = 83.0 Hz), 129.6, 128.9 (d, *J* = 12.4 Hz), 128.5 (d, *J* = 12.4 Hz), 127.8, 126.1, 35.3 (dd, *J* = 10.5, 6.7 Hz); ^{31}P NMR (162 MHz, $CDCl_3$): δ 49.8 (d, J_{P-P} = 54.2 Hz), 19.4 (d, J_{P-P} = 54.2 Hz); IR (KBr, cm^{-1}): 3054, 2923, 1587, 1436, 1206, 1109, 741, 718, 692, 528; HRMS (ESI+) *m/z* calcd for $C_{33}H_{28}NaOP_2S$ $[M+Na]^+$: 557.1234, found: 557.1228.

(*E*)-1-(diphenylphosphinyl)-2-(diphenylthiophosphinyl)-(2-cyclohexyl)ethene (3j)

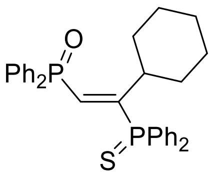

Colorless oil; ^1H NMR (400 MHz, $CDCl_3$): δ 7.83-7.75 (m, 4H), 7.63-7.56 (m, 4H), 7.53-7.48 (m, 4H), 7.47-7.39 (m, 8H), 6.82 (dd, J_{H-P} = 30.4, 21.3 Hz, 1H), 3.02-2.91 (m, 1H), 2.28-2.18 (m, 2H), 1.65-1.46 (m, 3H), 1.39-1.30 (m, 2H), 1.28-1.18 (m, 1H), 1.03-0.92 (m, 2H); ^{13}C NMR (100 MHz, $CDCl_3$): δ 161.3 (d, J_{C-P} = 56.3 Hz), 134.6 (dd, J_{C-P} = 87.7, 8.6 Hz), 134.1 (d, *J* = 105.9 Hz), 132.3 (d, *J* = 10.5 Hz), 132.9 (dd, *J* = 13.4, 1.9 Hz), 131.1 (d, *J* = 83.0 Hz),

130.8 (d, J = 9.5 Hz), 128.7 (dd, J = 12.4, 6.7 Hz), 43.1 (dd, J = 11.4, 5.7 Hz), 32.6, 29.4, 25.2; ^{31}P NMR (162 MHz, $CDCl_3$): δ 51.1 (d, $J_{P\text{-}P}$ = 60.6 Hz), 18.2 (d, $J_{P\text{-}P}$ = 60.6 Hz); IR (KBr, cm^{-1}): 3055, 2927, 2852, 2226, 1436, 1200, 1117, 1102, 734, 720, 694, 645; HRMS (ESI+) *m/z* calcd for $C_{32}H_{32}NaOP_2S$ $[M+Na]^+$: 549.1547, found: 549.1547.

(*E*)-1-(diphenylphosphinyl)-2-(diphenylthiophosphinyl)-2-(4-florophenyl)ethene (3k)

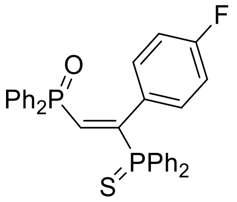

Colorless oil; ^{1}H NMR (400 MHz, $CDCl_3$): δ 7.78-7.73 (m, 4H), 7.56 (dd, $J_{H\text{-}P}$ = 24.9, 20.1 Hz, 1H), 7.53-7.44 (m, 5H), 7.40-7.35 (m, 6H), 7.28-7.24 (m, 5H), 6.90 (td, J = 6.9, 1.4 Hz, 2H), 6.51 (t, J = 8.7 Hz, 2H); ^{13}C NMR (100 MHz, $CDCl_3$): δ 162.7 (d, $J_{C\text{-}F}$ = 248.9 Hz), 156.2 (d, $J_{C\text{-}P}$ = 60.1 Hz), 138.3 (d, $J_{C\text{-}P}$ = 88.7, 9.5 Hz), 132.5 (d, $J_{C\text{-}P}$ = 105.9 Hz), 132.6 (d, $J_{C\text{-}P}$ = 10.5 Hz), 132.1 (d, J = 2.9 Hz), 130.0 (t, J = 4.8 Hz), 131.8 (d, J = 1.9 Hz), 130.9, 130.7 (d, J = 9.5 Hz), 129.5 (d, $J_{C\text{-}P}$ = 84.9 Hz), 128.57, 128.58 (d, J = 21.9 Hz), 128.0 (d, J = 12.4 Hz), 127.6 (d, J = 12.4 Hz), 114.4 (d, $J_{C\text{-}F}$ = 21.0 Hz); ^{19}F NMR (377 MHz, $CDCl_3$): δ -112.43; ^{31}P NMR (162 MHz, $CDCl_3$): δ 47.8 (d, $J_{P\text{-}P}$ = 50.7 Hz), 18.4 (d, $J_{P\text{-}P}$ = 50.7 Hz); IR (KBr, cm^{-1}): 3057, 1600, 1503, 1436, 1229, 1187, 1099, 838, 747, 726, 692, 639, 548, 528, 503; HRMS (ESI+) *m/z* calcd for $C_{32}H_{25}FNaOP_2S$ $[M+Na]^+$: 561.0983, found: 561.0982.

(*E*)-1-(diphenylphosphinyl)-2-(diphenylthiophosphinyl)-2-(4-methoxyphenyl)ethene (3l)

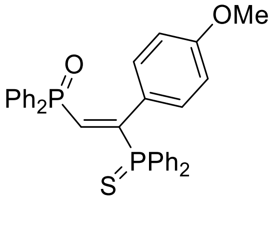

Colorless oil; ^{1}H NMR (400 MHz, $CDCl_3$): δ 7.77-7.72 (m, 4H), 7.58 (dd, J_{H-P} = 25.4, 20.1 Hz, 1H), 7.56-7.51 (m, 4H), 7.50-7.45 (m, 2H), 7.41-7.36 (m, 6H), 7.32-7.27 (m, 4H), 6.85 (dd, *J* = 8.7, 1.8 Hz, 2H), 6.39 (d, *J* = 8.2 Hz, 2H), 3.65 (s, 3H); ^{13}C NMR (100 MHz, $CDCl_3$): δ 159.4, 152.6 (d, J_{C-P} = 59.4 Hz), 138.9 (dd, J_{C-P} = 73.3, 11.0 Hz), 132.9 (d, *J* = 105.9 Hz), 132.7 (d, *J* = 10.5 Hz), 131.63 (d, *J* = 2.9 Hz), 131.57 (d, *J* = 3.8 Hz), 132.0 (d, *J* = 2.9 Hz), 131.9, 131.7 (dd, *J* = 8.6, 2.9 Hz), 131.4, 131.3, 130.2 (d, *J* = 83.4 Hz), 128.6 (d, *J* = 12.5 Hz), 128.4 (d, *J* = 13.4 Hz), 124.8, 112.7, 55.2; ^{31}P NMR (162 MHz, $CDCl_3$): δ 47.5 (d, J_{P-P} = 52.0 Hz), 17.90 (d, J_{P-P} = 52.0 Hz); IR (KBr, cm^{-1}): 3053, 2924, 1605, 1505, 1436, 1250, 1180, 1099, 748, 719, 693, 640; HRMS (ESI+) *m/z* calcd for $C_{33}H_{28}NaO_2P_2S$ $[M+Na]^+$: 573.1183, found: 573.1183.

(*E*)-1-(diphenylphosphinyl)-2-(diphenylthiophosphinyl)-2-(4-tert-butylphenyl)ethene (3m)

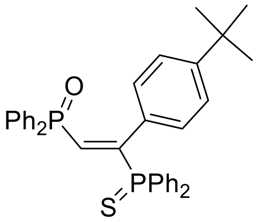

White solid; mp. > 250 °C; ^{1}H NMR (400 MHz, $CDCl_3$): δ 7.74-7.68 (m, 4H), 7.64 (dd, J_{H-P} = 25.7, 19.7 Hz, 1H), 7.53-7.44 (m, 6H), 7.39-7.32 (m, 6H), 7.28-7.24 (m, 4H), 6.83 (d, *J* = 8.2 Hz, 2H), 6.78 (dd, *J* = 8.7, 1.4 Hz, 2H), 1.41 (s, 9H); ^{13}C NMR (100 MHz, $CDCl_3$): δ 156.8 (d, J_{C-P} = 70.9 Hz), 151.3, 138.2 (dd, J_{C-P} = 89.1, 8.6 Hz), 133.0 (d, *J* = 106.4 Hz), 132.7 (d, *J* = 10.54 Hz), 132.0 (d, *J* = 2.9 Hz), 131.5 (d, *J* = 1.9 Hz), 130.8 (d, *J* = 9.6 Hz), 130.1 (d, *J* = 84.35 Hz), 129.9 (d, *J* = 3.8 Hz), 128.5 (dd, *J* = 12.5, 7.7 Hz), 124.2, 34.5, 31.2; ^{31}P NMR (162 MHz, $CDCl_3$): δ 48.5 (d, J_{P-P} = 52.0 Hz), 18.9 (d, J_{P-P} = 52.0 Hz); IR (KBr) 3051, 2959, 1607, 1548, 1479, 1437, 847, 750, 715, 691, 644; HRMS (ESI+) *m/z* calcd for $C_{36}H_{34}NaOP_2S$ $[M+Na]^+$: 599.1703, found: 599.1702.

(*E*)-1-(diphenylphosphinyl)-2-(diphenylthiophosphinyl)-2-(4-octylphenyl)ethene (3n)

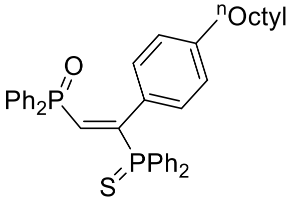

Colorless oil; ^{1}H NMR (400 MHz, $CDCl_3$): δ 7.74-7.68 (m, 4H), 7.66 (dd, $J_{H\text{-}P}$ = 25.4, 20.2 Hz, 1H), 7.55-7.49 (m, 4H), 7.46-7.43 (m, 2H), 7.38-7.33 (m, 6H), 7.29-7.24 (m, 4H), 6.76 (dd, *J* = 7.8, 1.4 Hz, 2H), 6.63 (d, *J* = 7.8 Hz, 2H), 2.36 (t, *J* = 7.6 Hz, 2H), 1.46-1.36 (m, 2H), 1.31-1.21 (m, 10H), 0.88 (t, *J* = 6.6 Hz, 3H); ^{13}C NMR (100 MHz, $CDCl_3$): δ 156.9 (d, *J* = 58.5 Hz), 143.4, 138.0 (dd, $J_{C\text{-}P}$ = 89.1, 9.6 Hz), 133.0 (d, $J_{C\text{-}P}$ = 106.4 Hz), 132.7 (d, *J* = 9.6 Hz), 131.9 (d, *J* = 1.9 Hz), 131.5 (d, *J* = 2.9 Hz), 130.8 (d, *J* = 10.5 Hz), 130.6 (d, *J* = 8.6 Hz), 129.99 (d, *J* = 84.4 Hz), 129.95 (d, *J* = 3.8 Hz), 128.4 (dd, *J* = 12.5, 6.7 Hz), 127.3, 35.6, 32.0, 31.3, 29.5, 29.4, 29.3, 22.8, 14.2; ^{31}P NMR (162 MHz, $CDCl_3$): δ 48.4 (d, $J_{P\text{-}P}$ = 52.0 Hz), 18.9 (d, $J_{P\text{-}P}$ = 52.0 Hz); IR (KBr, cm^{-1}): 2925, 2854, 1437, 1186, 1106, 720, 693; HRMS (ESI+) *m/z* calcd for $C_{40}H_{42}NaOP_2S$ $[M+Na]^+$: 655.2329, found: 655.2329.

(*E*)-1-(diphenylphosphinyl)-2-(diphenylthiophosphinyl)-2-(4-diphenylyl)ethene (3o)

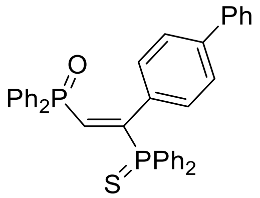

White solid; mp. 89-90 °C; ^{1}H NMR (400 MHz, $CDCl_3$): δ 7.80-7.75 (m, 4H), 7.68 (dd, $J_{H\text{-}P}$ = 30.2, 19.2 Hz, 1H), 7.56-7.52 (m ,4H), 7.45-7.47 (m, 2H), 7.43-7.39 (m, 8H), 7.30-7.27 (m, 7H), 7.06 (d, *J* = 8.2 Hz, 2H), 6.93 (dd, *J* = 8.2, 1.4 Hz, 2H); ^{13}C NMR (100 MHz, $CDCl_3$): δ 152.3 (d, $J_{C\text{-}P}$ = 59.1 Hz), 141.0 (dd, $J_{C\text{-}P}$ = 72.5, 9.5 Hz), 132.8 (d, *J* = 10.5 Hz), 132.7 (d, *J* = 4.8 Hz), 132.4, 132.3 (d, *J* = 5.7 Hz), 132.1, 131.8, 131.7 (d, *J* = 78.2 Hz), 131.6 (d, *J* = 8.6 Hz), 131.5 (d, *J* = 10.5 Hz), 131.3 (d, *J* = 2.9 Hz), 130.8 (d, *J* = 2.9 Hz), 130.2 (d, *J* = 84.9 Hz),

128.9, 128.6 (d, J = 12.4 Hz), 128.5 (d, J = 12.4 Hz), 128.4 (d, J = 13.4 Hz), 127.6, 127.0, 126.8 (d, J = 68.6 Hz), 125.8; ^{31}P NMR (162 MHz, $CDCl_3$): δ 48.4 (d, J_{P-P} = 52.0 Hz), 18.9 (d, J_{P-P} = 52.0 Hz); IR (KBr, cm^{-1}): 3053, 1482, 1436, 1185, 1099, 719, 692, 642, 545, 509; HRMS (ESI+) *m/z* calcd for $C_{40}H_{42}NaOP_2S$ $[M+Na]^+$: 655.2329, found: 655.2329.

2. Copies of NMR spectra

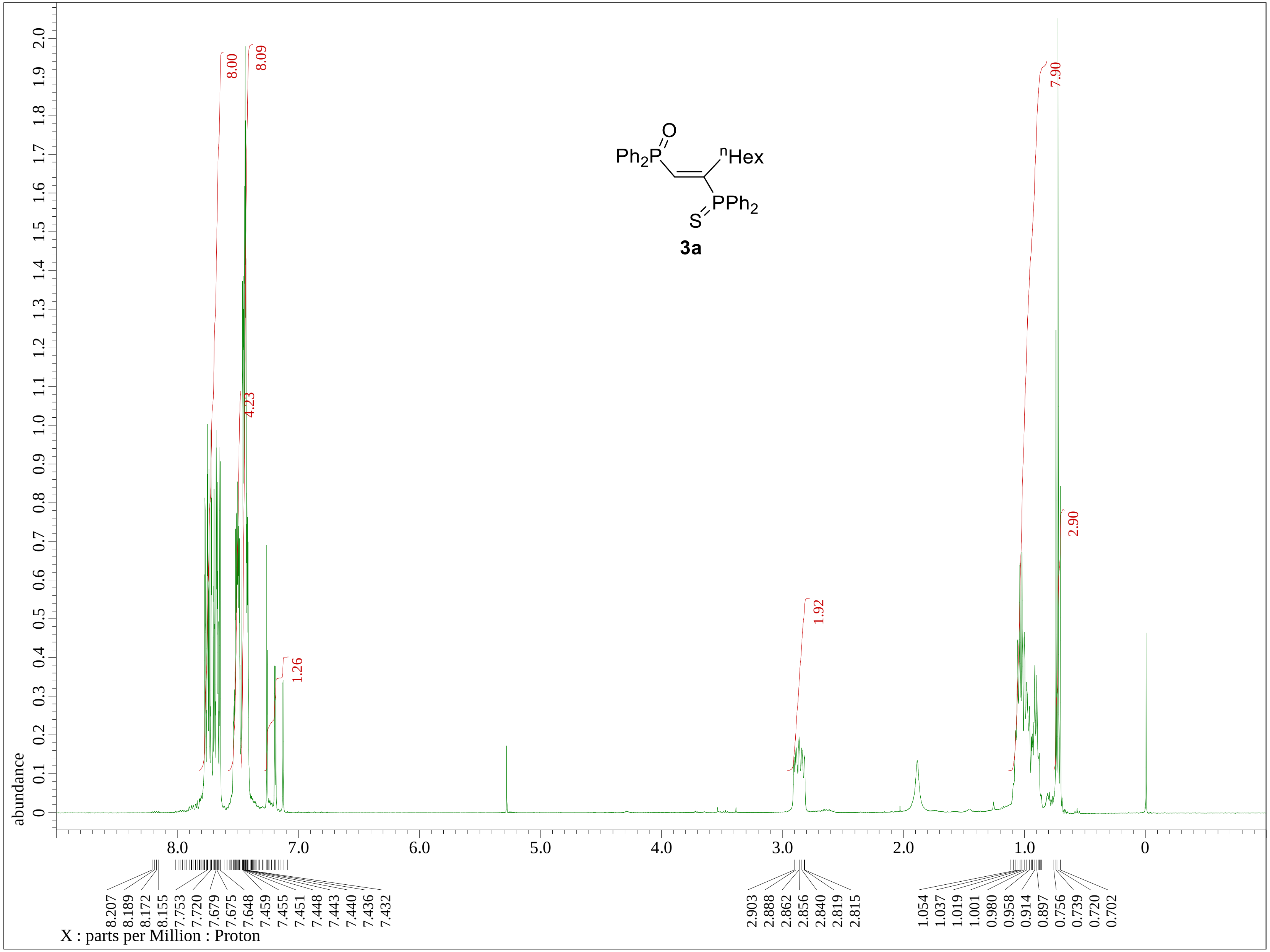

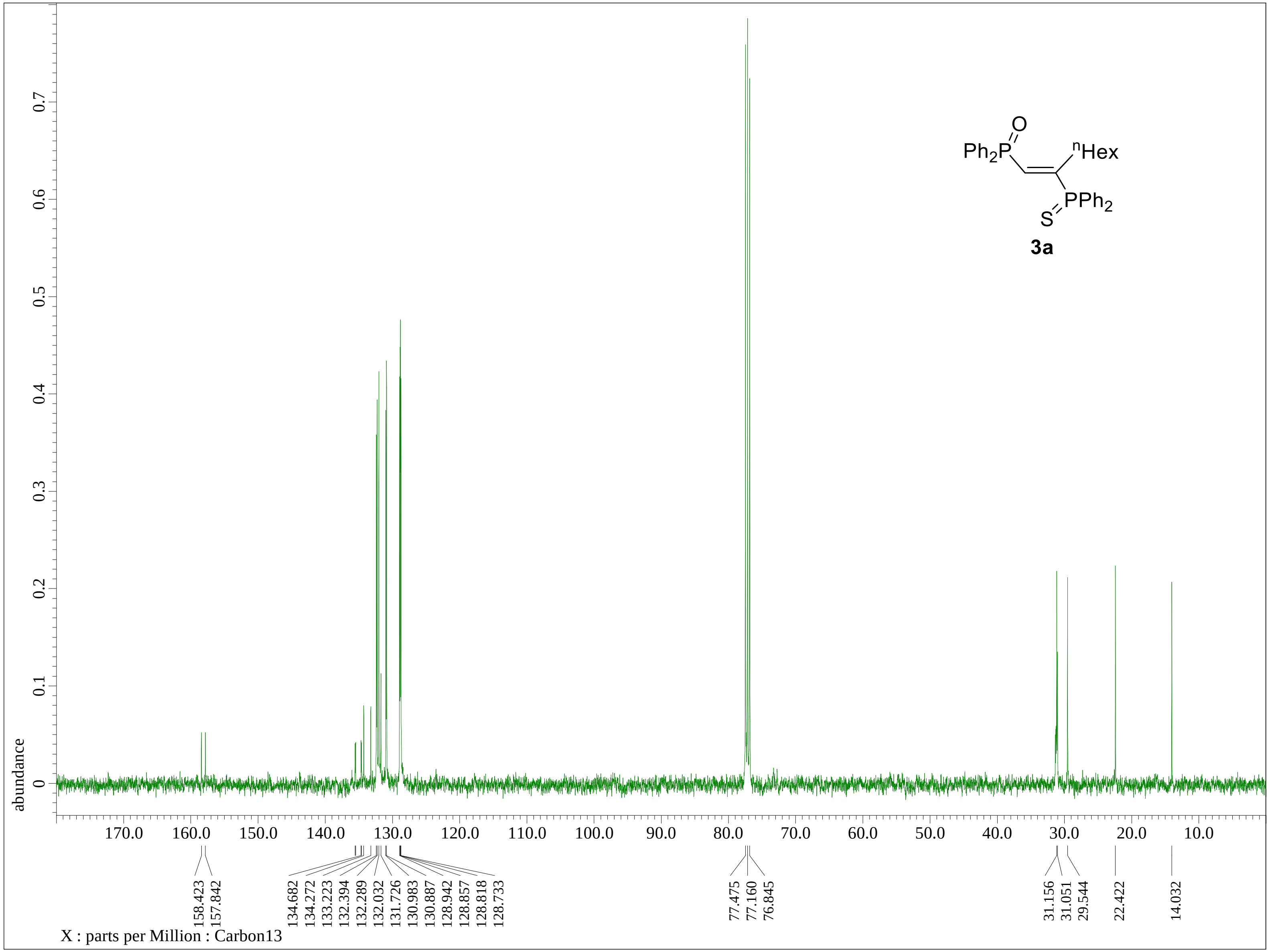

abundance
0.7
0.6
0.5
0.4
0.3
0.2
0.1
0
170.0
160.0
150.0
140.0
130.0
120.0
110.0
100.0
90.0
80.0
70.0
60.0
50.0
40.0
30.0
20.0
10.0
158.423
157.842
134.682
134.272
133.223
132.394
132.289
132.032
131.726
130.983
130.887
128.942
128.857
128.818
128.733
77.475
77.160
76.845
31.156
31.051
29.544
22.422
14.032
X : parts per Million : Carbon13
Ph2P
O
nHex
PPh2
S
3a

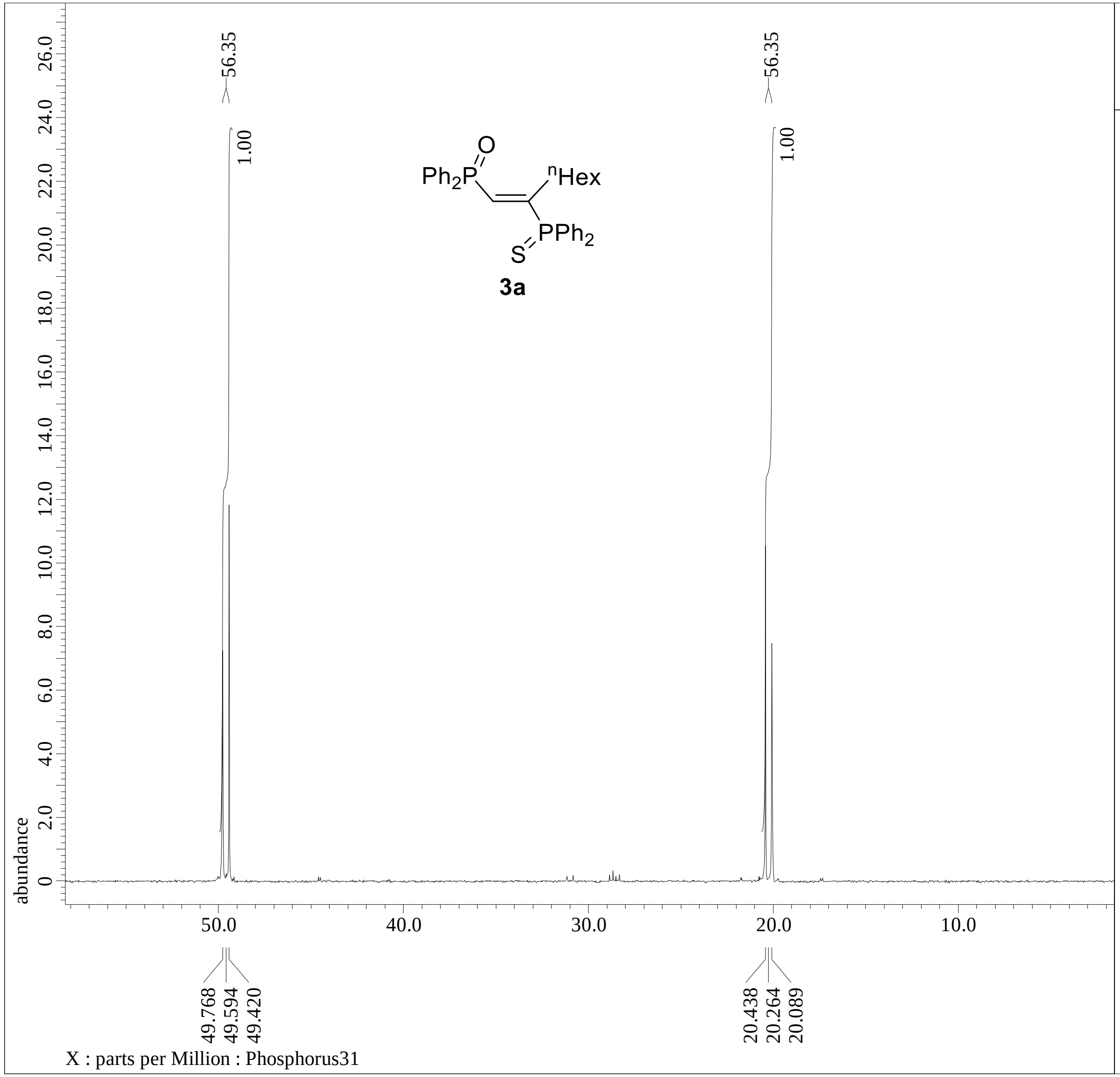


```
Filename          = C:\Documents and Setting
Author            = delta
Experiment        = carbon.jxp
Sample_Id         = Dat 0626HexPPO GPC
Solvent           = CHLOROFORM-D
Creation_Time     = 26-JUN-2020 14:01:15
Revision_Time     = 26-JUN-2020 14:32:41
Current_Time      = 26-JUN-2020 14:32:51

Comment           = single pulse decoupled g
Data_Format       = 1D COMPLEX
Dim_Size          = 26214
Dim_Title         = Phosphorus31
Dim_Units         = [ppm]
Dimensions        = X
Site              = ECX 400
Spectrometer      = DELTA2_NMR

Field_Strength    = 9.389766[T] (400[MHz])
X_Acq_Duration    = 0.23068672[s]
X_Domain          = 31P
X_Freq            = 161.83469309[MHz]
X_Offset          = 0[ppm]
X_Points          = 32768
X_Prescans        = 4
X_Resolution      = 4.33488326[Hz]
X_Sweep           = 142.04545455[kHz]
X_Sweep_Clipped   = 113.63636364[kHz]
Irr_Domain        = Proton
Irr_Freq          = 399.78219838[MHz]
Irr_Offset        = 5[ppm]
Clipped           = FALSE
Scans             = 64
Total_Scans       = 64

Relaxation_Delay  = 2[s]
Recvr_Gain        = 50
Temp_Get          = 26.9[dC]
X_90_Width        = 14[us]
X_Acq_Time        = 0.23068672[s]
X_Angle           = 30[deg]
X_Atn             = 9[dB]
X_Pulse           = 4.66666667[us]
Irr_Atn_Dec       = 23.2[dB]
Irr_Atn_Noe       = 23.2[dB]
Irr_Noise         = WALTZ
Irr_Pwidth        = 0.115[ms]
Decoupling        = TRUE
Initial_Wait      = 1[s]
Noe               = TRUE
Noe_Time          = 2[s]
Repetition_Time   = 2.23068672[s]
```

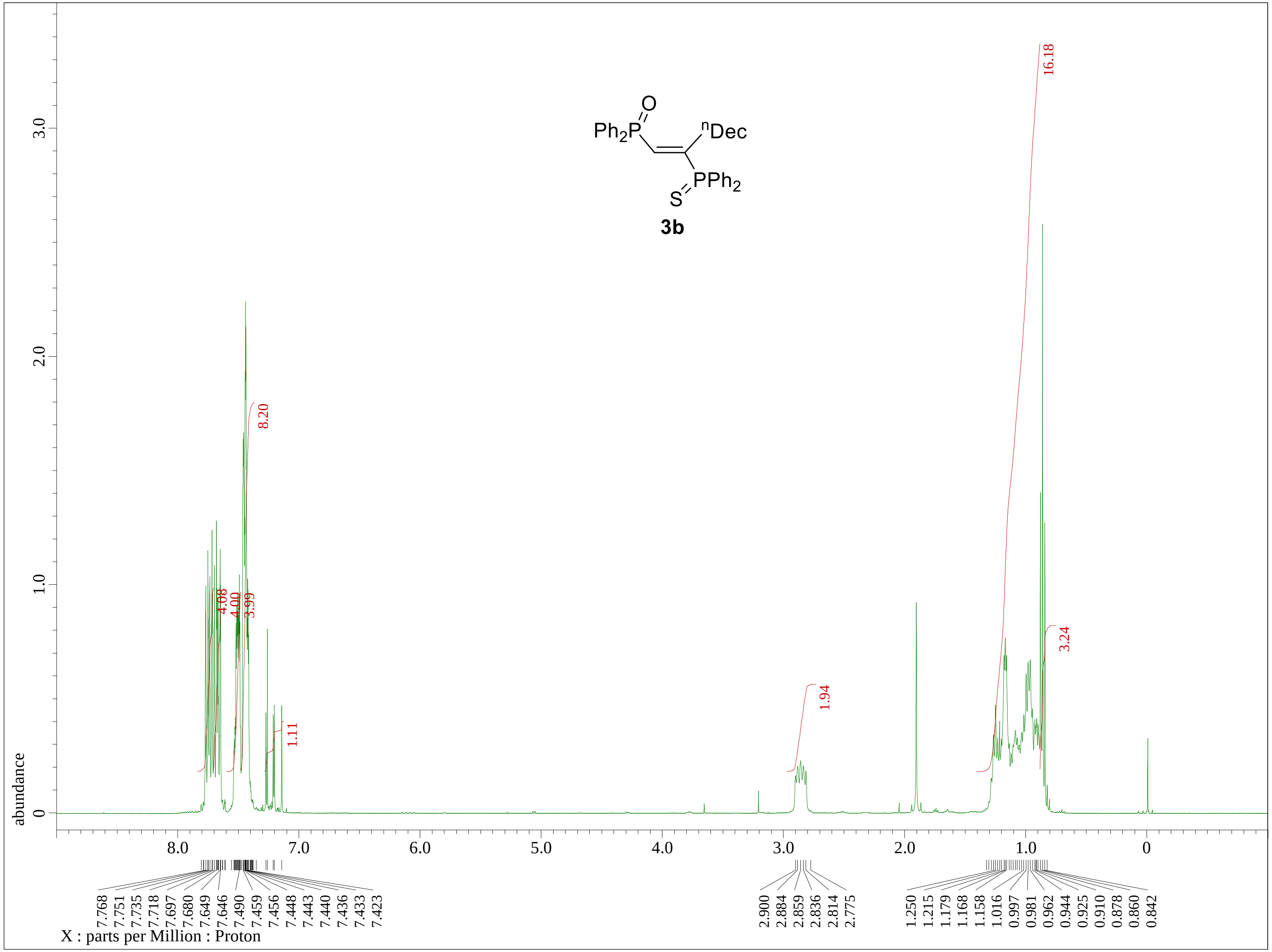

Ph2P
O
nDec
PPh2
S
3b
abundance
X : parts per Million : Proton

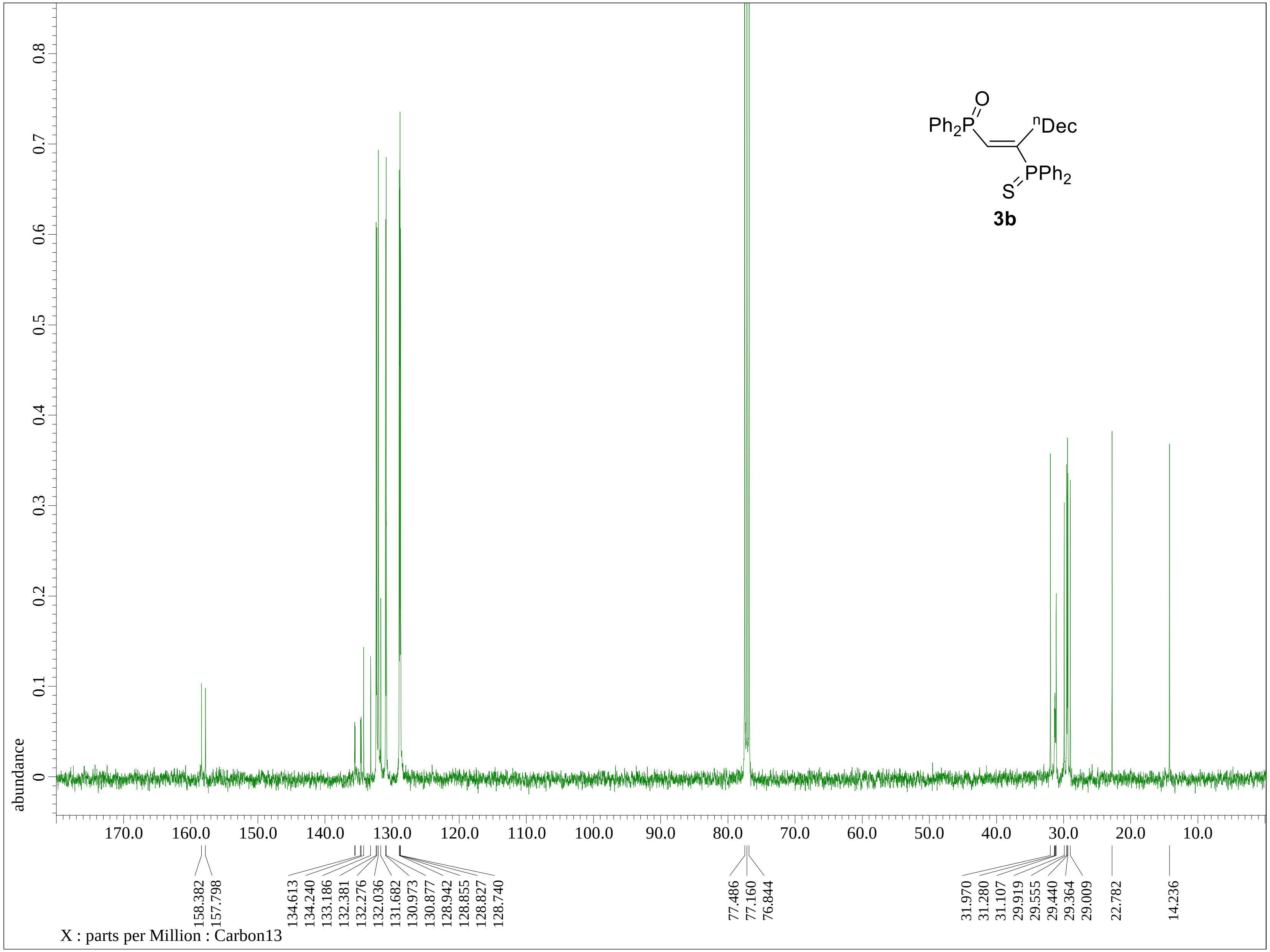

Ph2P
O
nDec
PPh2
S
3b
abundance
X : parts per Million : Carbon13
158.382
157.798
134.613
134.240
133.186
132.381
132.276
132.036
131.682
130.973
130.877
128.942
128.855
128.827
128.740
77.486
77.160
76.844
31.970
31.280
31.107
29.919
29.555
29.440
29.364
29.009
22.782
14.236

"D 0918 D6 TC2" 1 1 D:\nmrdata\OGAWA

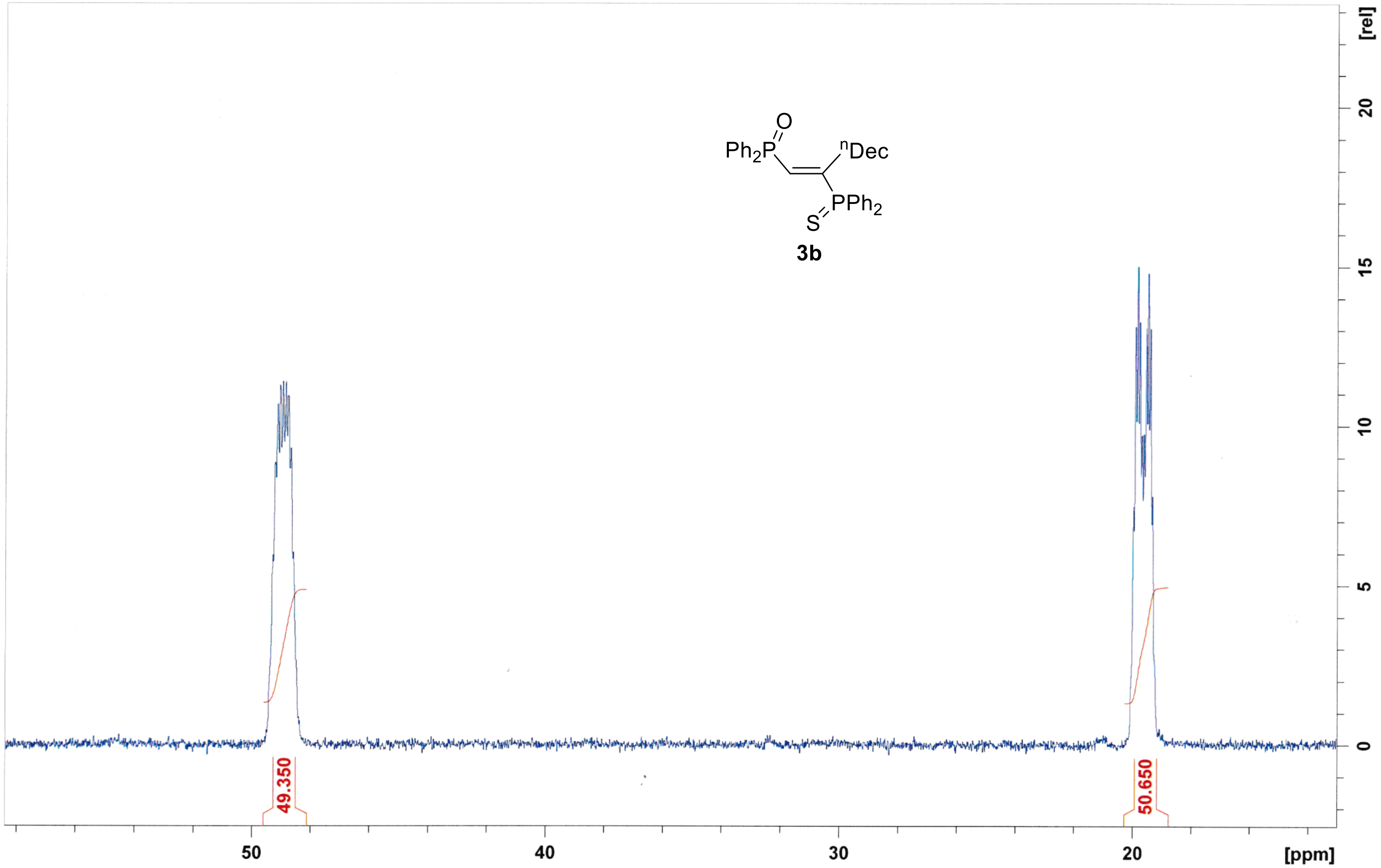

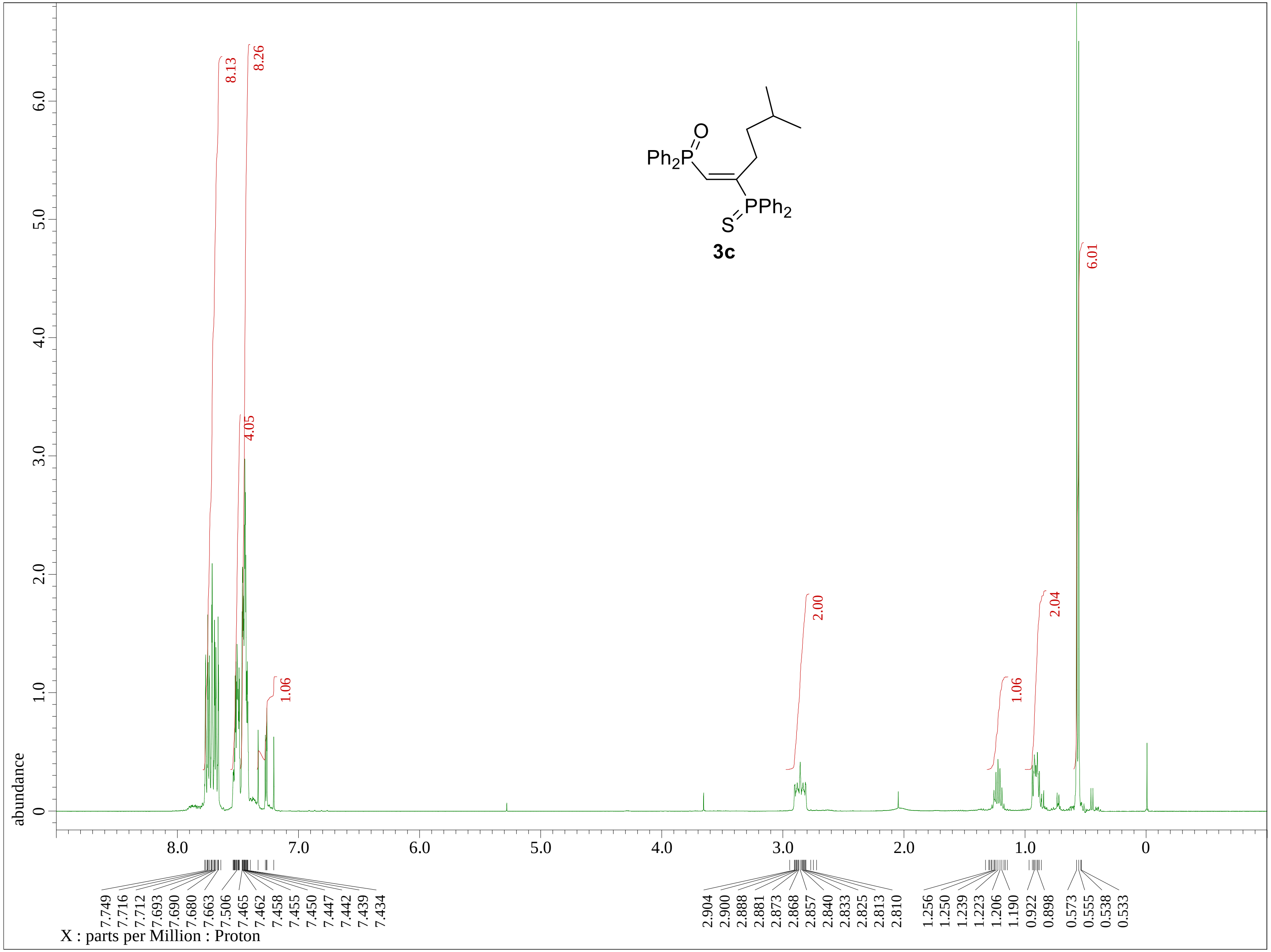

Ph2P=O
S=PPh2
3c
8.13
8.26
4.05
1.06
2.00
1.06
2.04
6.01
abundance
0
1.0
2.0
3.0
4.0
5.0
6.0
8.0
7.0
6.0
5.0
4.0
3.0
2.0
1.0
0
7.749
7.716
7.712
7.693
7.690
7.680
7.663
7.506
7.465
7.462
7.458
7.455
7.450
7.447
7.442
7.439
7.434
2.904
2.900
2.888
2.881
2.873
2.868
2.857
2.840
2.833
2.825
2.813
2.810
1.256
1.250
1.239
1.223
1.206
1.190
0.922
0.898
0.573
0.555
0.538
0.533
X : parts per Million : Proton

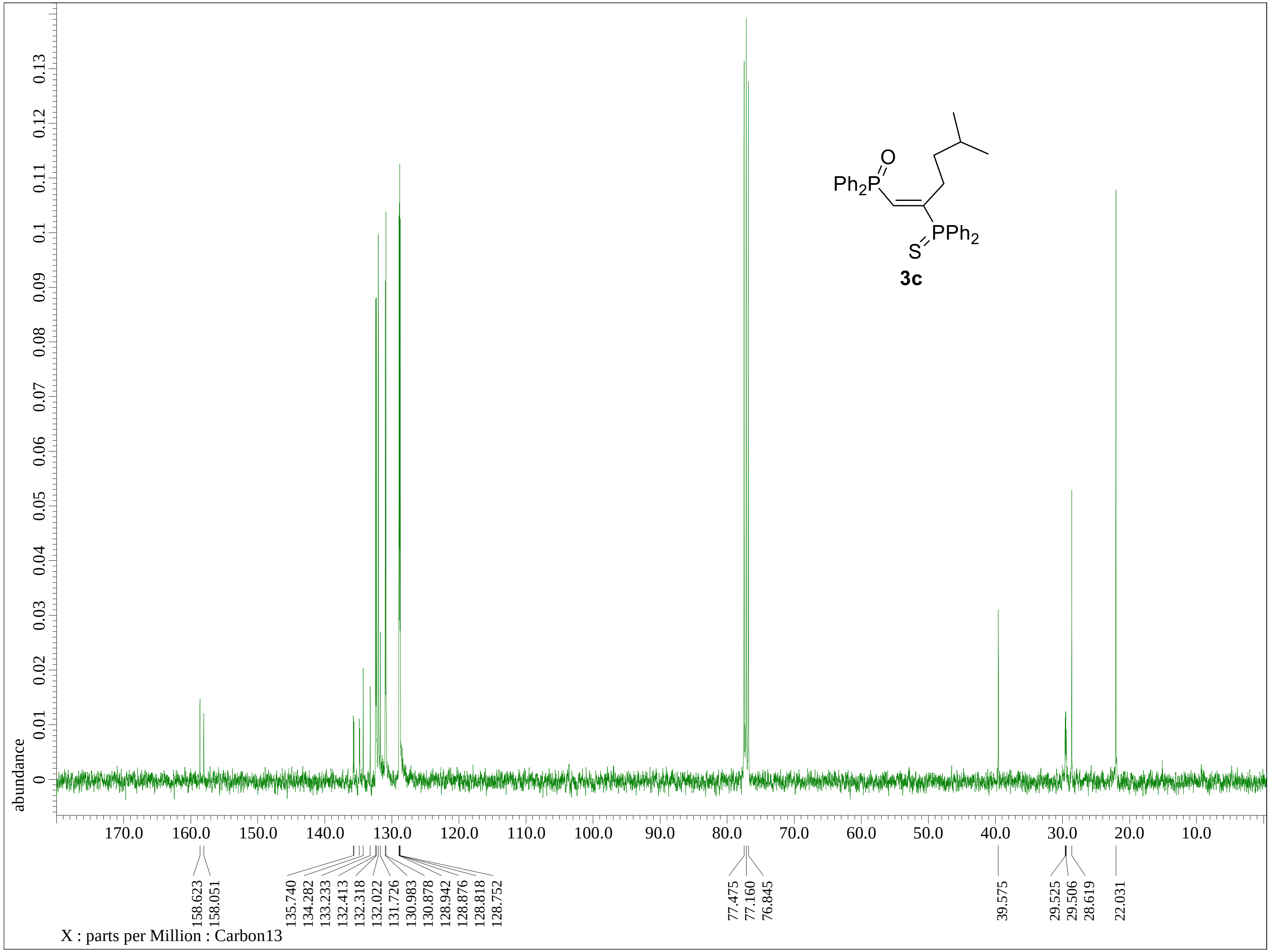

Ph2P=O
S=PPh2
3c
abundance
0
0.01
0.02
0.03
0.04
0.05
0.06
0.07
0.08
0.09
0.1
0.11
0.12
0.13
170.0
160.0
150.0
140.0
130.0
120.0
110.0
100.0
90.0
80.0
70.0
60.0
50.0
40.0
30.0
20.0
10.0
158.623
158.051
135.740
134.282
133.233
132.413
132.318
132.022
131.726
130.983
130.878
128.942
128.876
128.818
128.752
77.475
77.160
76.845
39.575
29.525
29.506
28.619
22.031
X : parts per Million : Carbon13

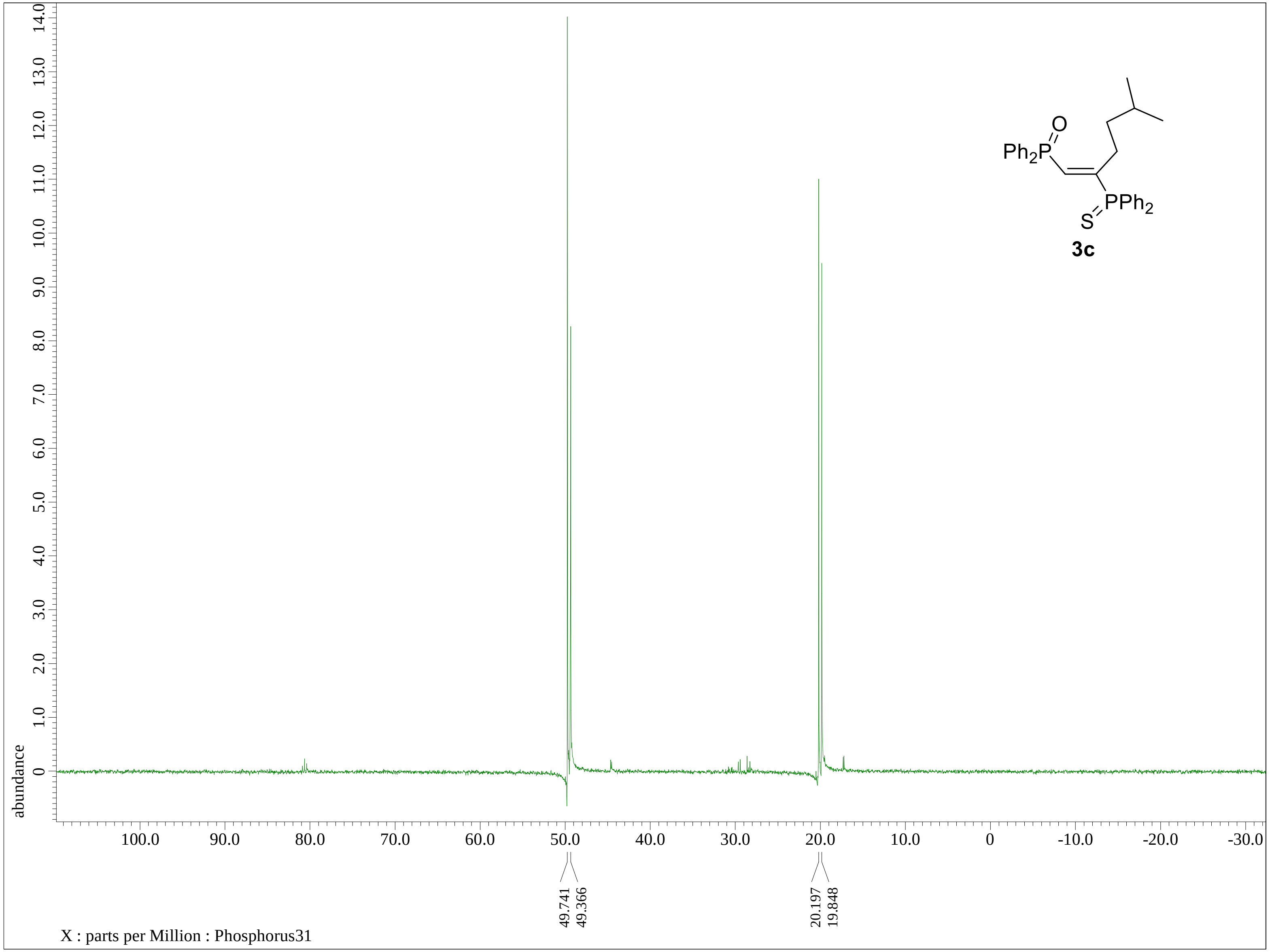

Ph2P
O
PPh2
S
3c
abundance
X : parts per Million : Phosphorus31
49.741
49.366
20.197
19.848

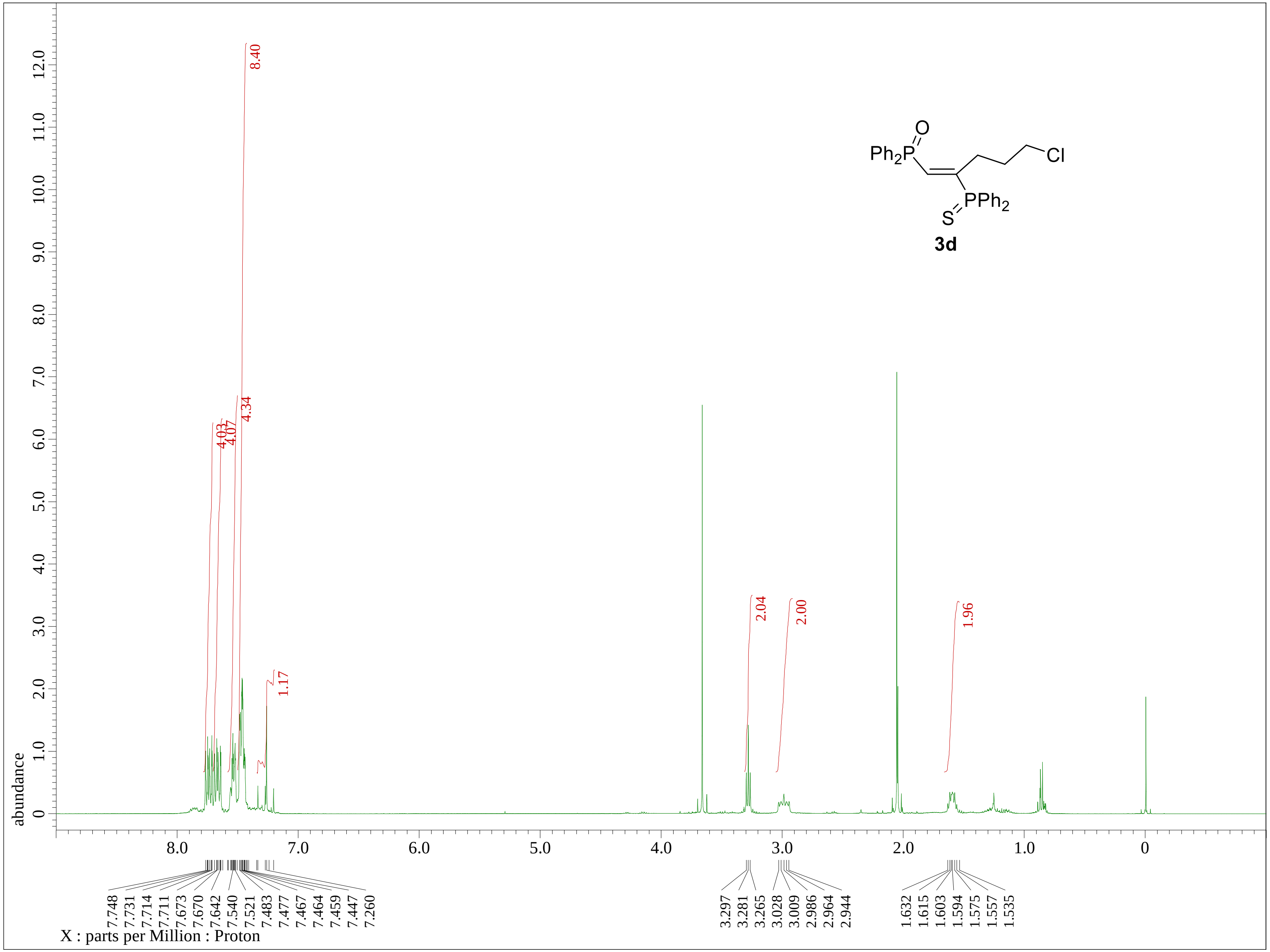

3d
abundance
X : parts per Million : Proton
8.40
4.34
4.03
4.07
1.17
2.04
2.00
1.96
7.748
7.731
7.714
7.711
7.673
7.670
7.642
7.540
7.521
7.483
7.477
7.467
7.464
7.459
7.447
7.260
3.297
3.281
3.265
3.028
3.009
2.986
2.964
2.944
1.632
1.615
1.603
1.594
1.575
1.557
1.535

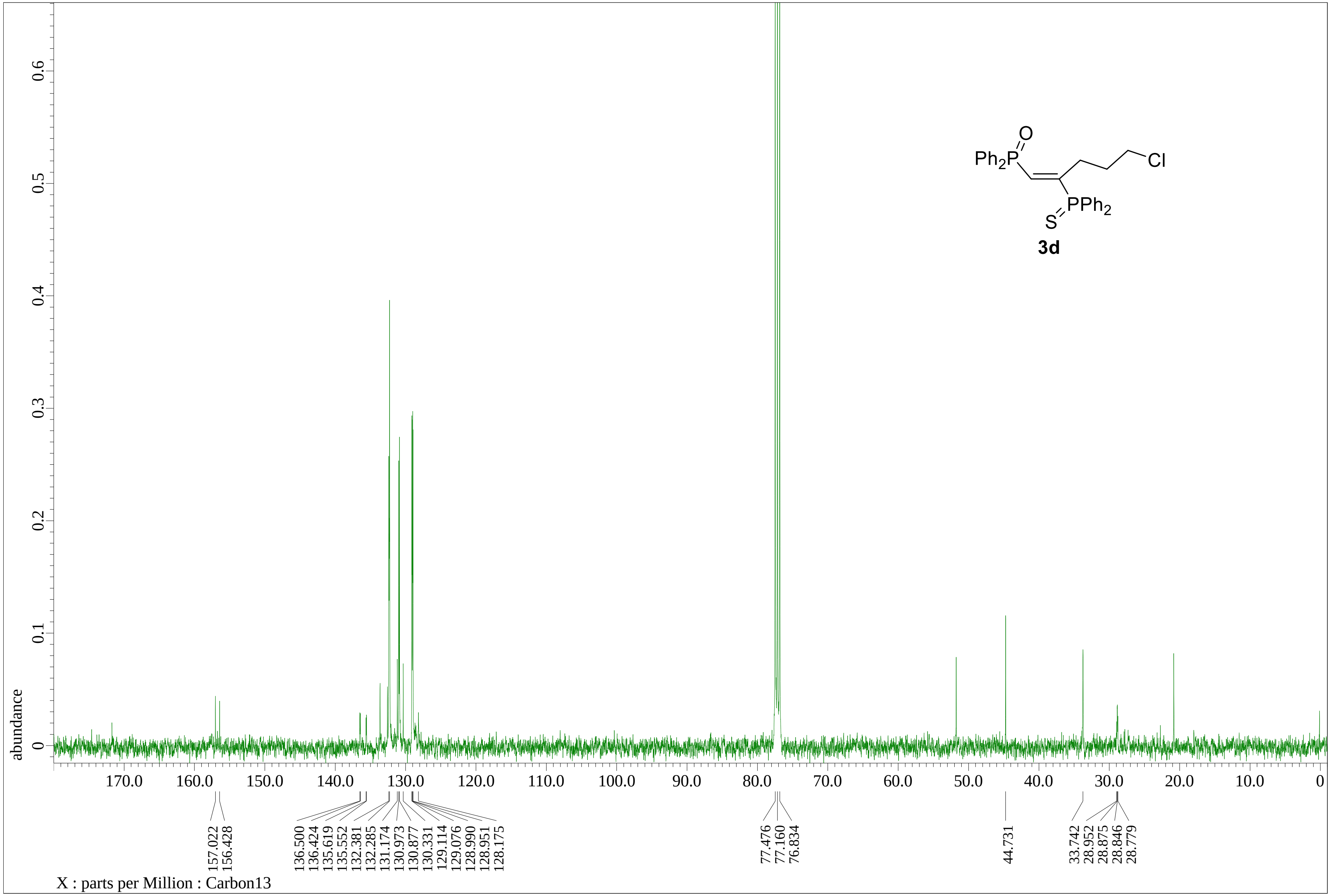
Ph2P
O
Cl
PPh2
S
3d
abundance
0
0.1
0.2
0.3
0.4
0.5
0.6
170.0
160.0
150.0
140.0
130.0
120.0
110.0
100.0
90.0
80.0
70.0
60.0
50.0
40.0
30.0
20.0
10.0
0
157.022
156.428
136.500
136.424
135.619
135.552
132.381
132.285
131.174
130.973
130.877
130.331
129.114
129.076
128.990
128.951
128.175
77.476
77.160
76.834
44.731
33.742
28.952
28.875
28.846
28.779
X : parts per Million : Carbon13

"D 0925 penclo S1" 1 1 D:\nmrdata\OGAWA

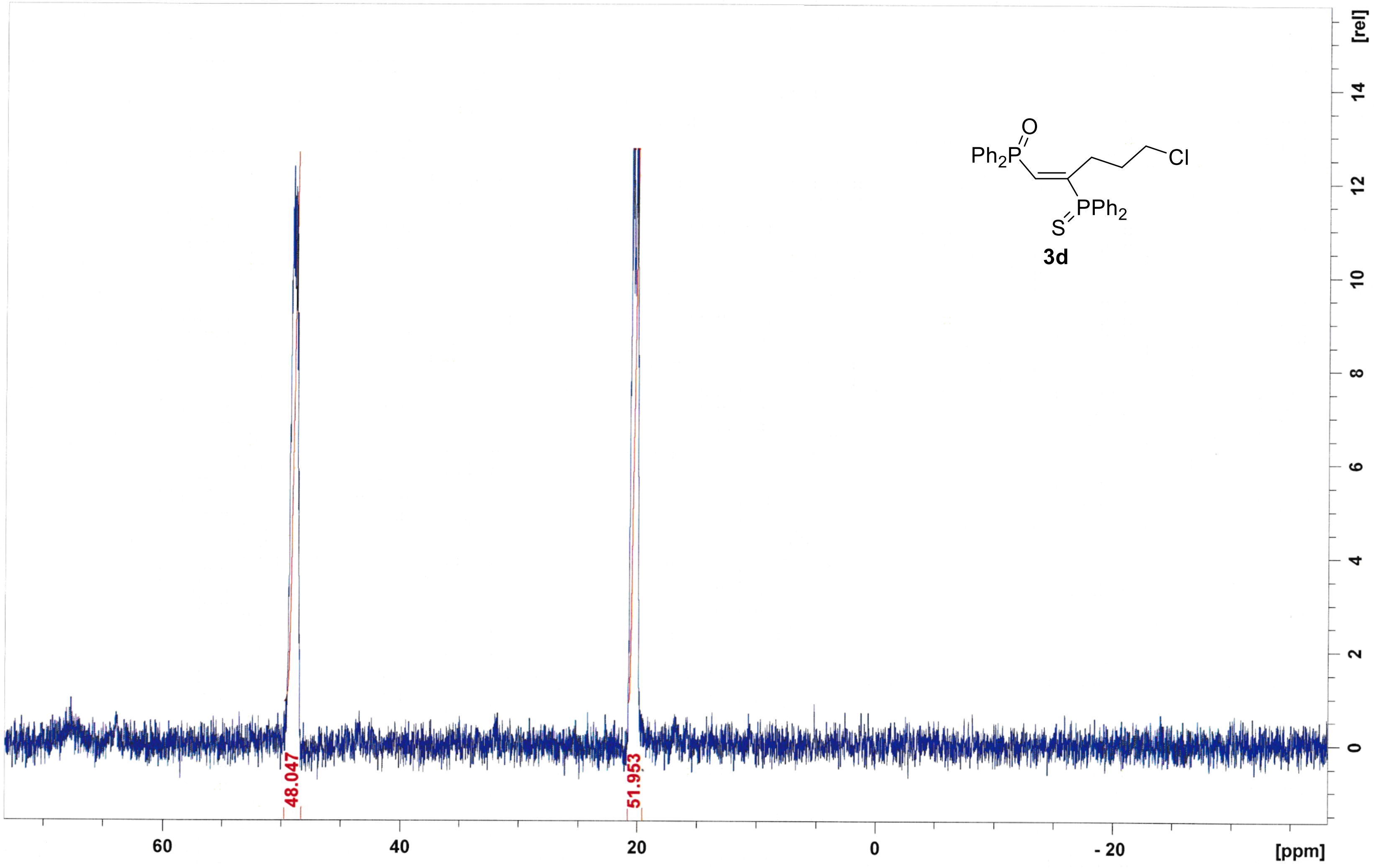

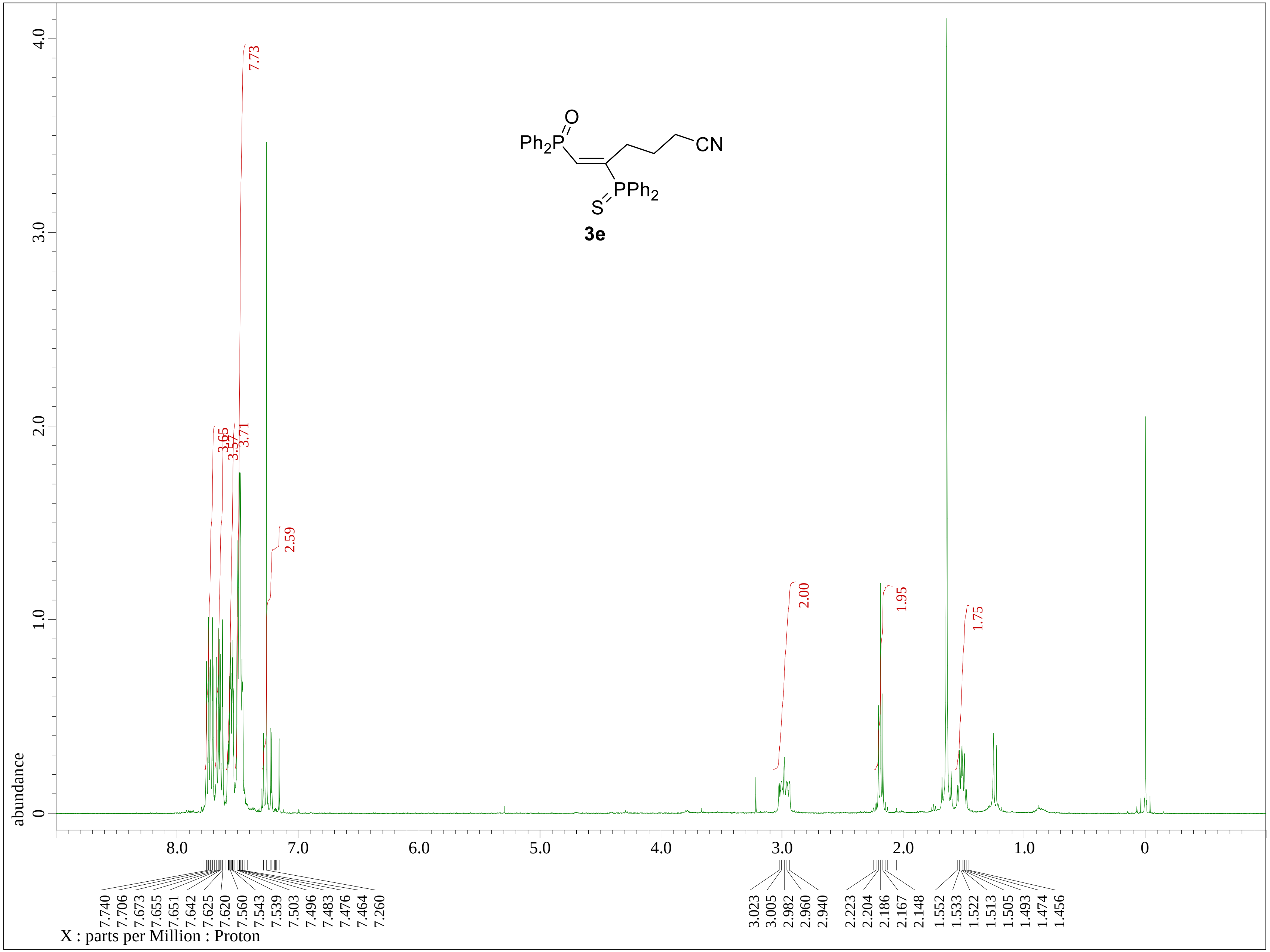
abundance
X : parts per Million : Proton
7.740
7.706
7.673
7.655
7.651
7.642
7.625
7.620
7.560
7.543
7.539
7.503
7.496
7.483
7.476
7.464
7.260
3.023
3.005
2.982
2.960
2.940
2.223
2.204
2.186
2.167
2.148
1.552
1.533
1.522
1.513
1.505
1.493
1.474
1.456
7.73
3.65
3.57
3.71
2.59
2.00
1.95
1.75
Ph2P
O
CN
PPh2
S

3e

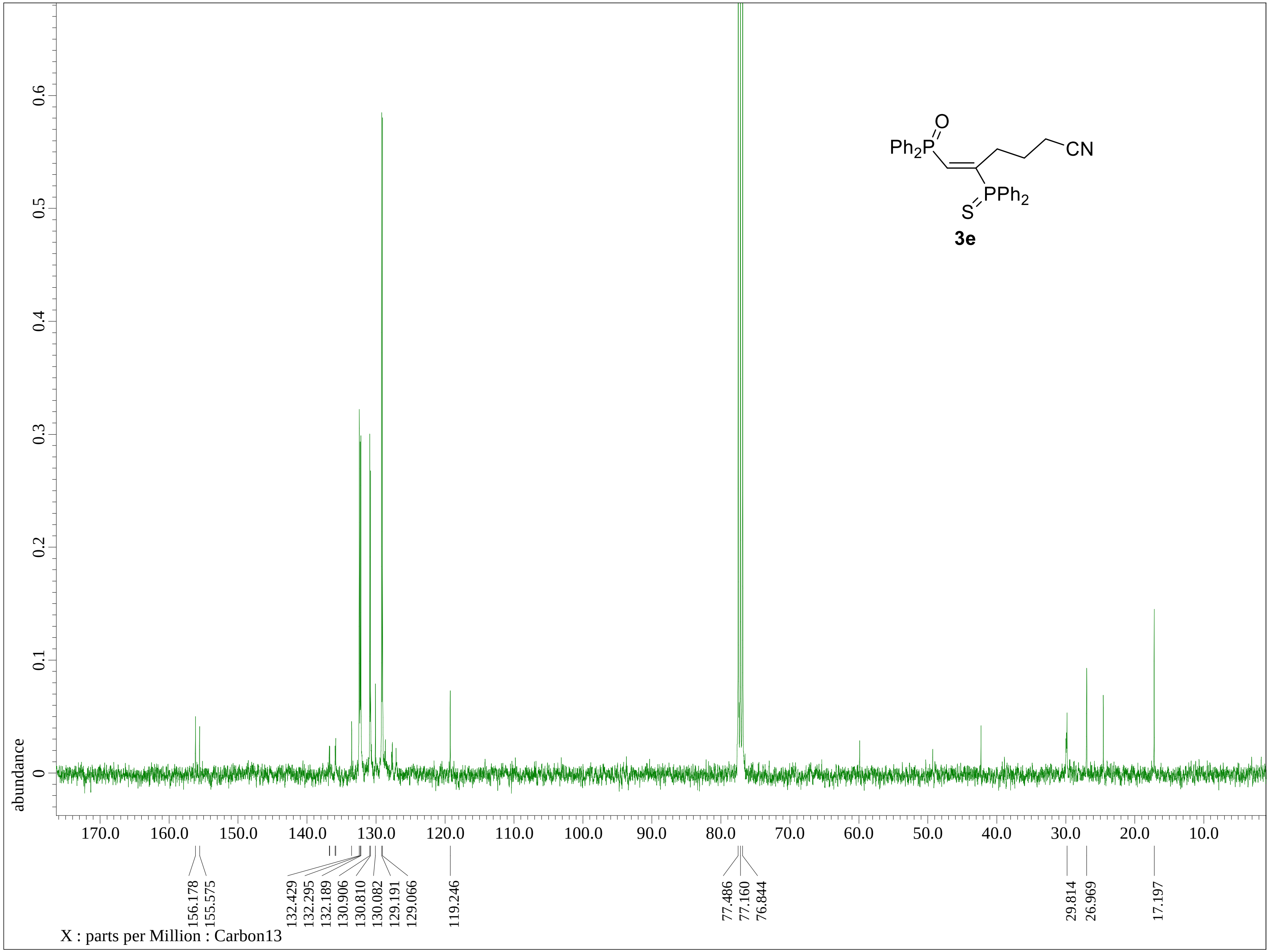
Ph2P
O
CN
PPh2
S
3e
abundance
0
0.1
0.2
0.3
0.4
0.5
0.6
170.0
160.0
150.0
140.0
130.0
120.0
110.0
100.0
90.0
80.0
70.0
60.0
50.0
40.0
30.0
20.0
10.0
156.178
155.575
132.429
132.295
132.189
130.906
130.810
130.082
129.191
129.066
119.246
77.486
77.160
76.844
29.814
26.969
17.197
X : parts per Million : Carbon13

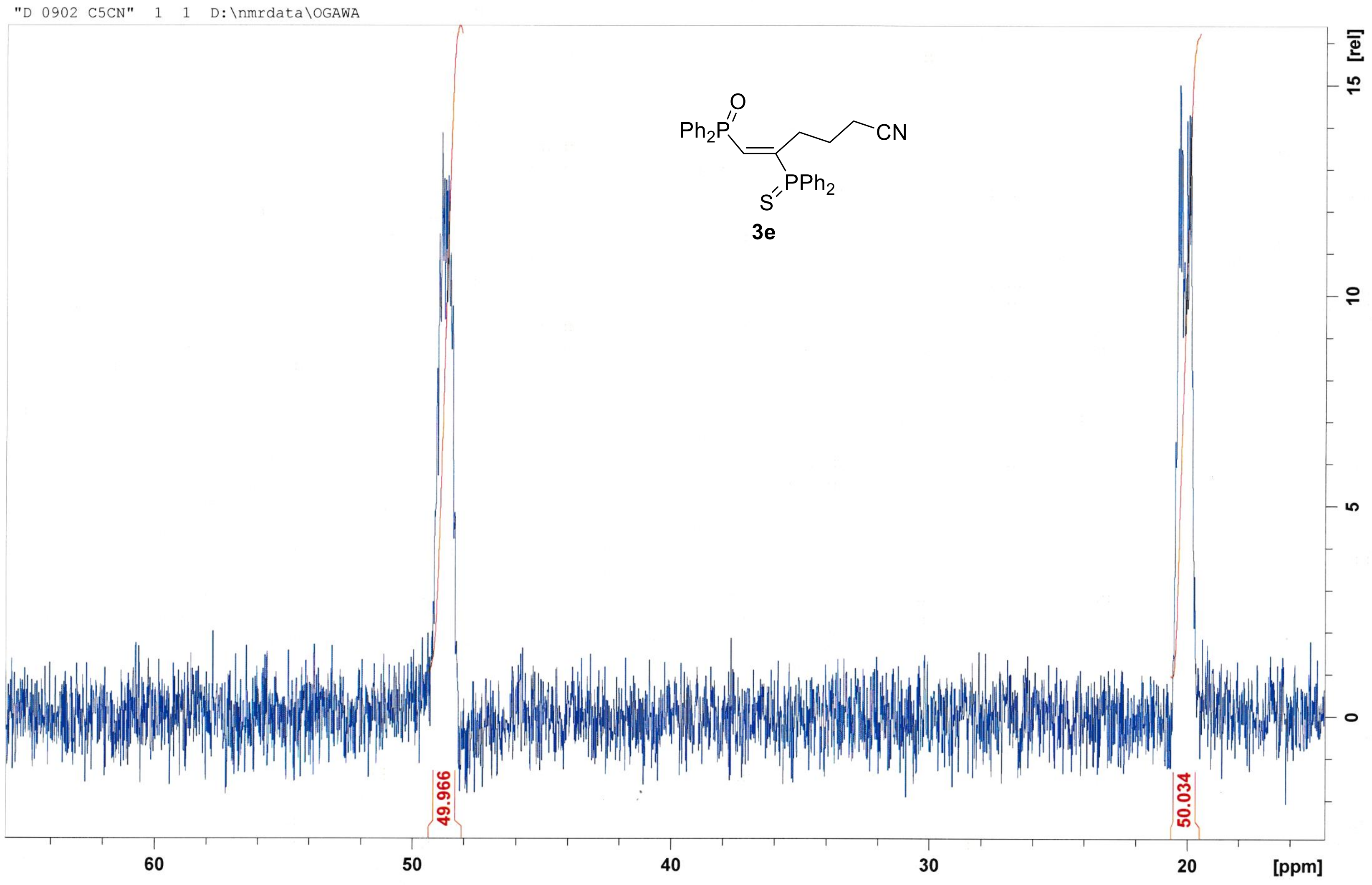

"D 0902 C5CN" 1 1 D:\nmrdata\OGAWA
Ph2P
O
CN
S
PPh2
3e
49.966
50.034
60
50
40
30
20
[ppm]
15
10
5
0
[rel]

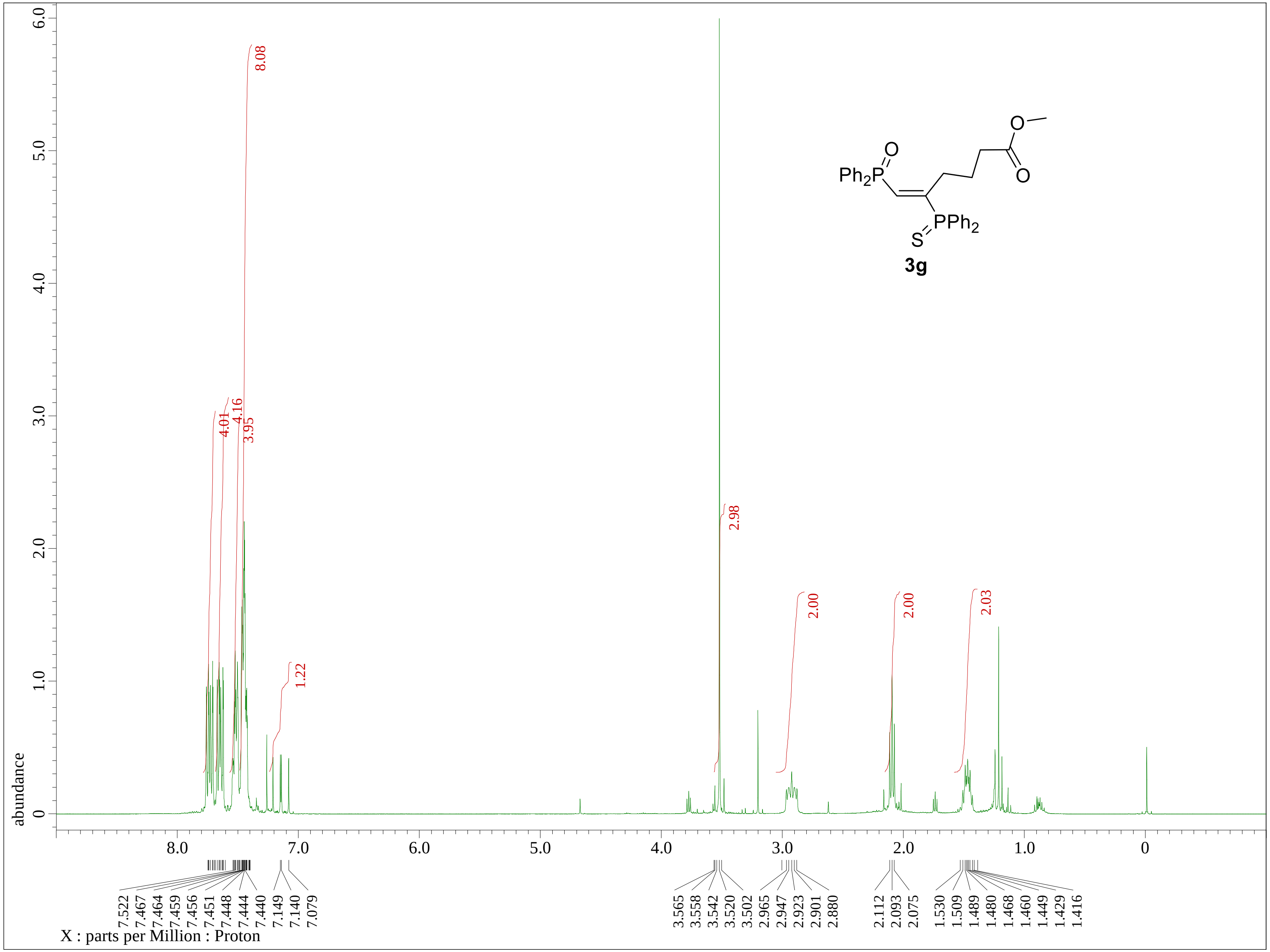
Ph2P
O
O
O
PPh2
S
3g
abundance
X : parts per Million : Proton
8.08
4.01
4.16
3.95
1.22
2.98
2.00
2.00
2.03
7.522
7.467
7.464
7.459
7.456
7.451
7.448
7.444
7.440
7.149
7.140
7.079
3.565
3.558
3.542
3.520
3.502
2.965
2.947
2.923
2.901
2.880
2.112
2.093
2.075
1.530
1.509
1.489
1.480
1.468
1.460
1.449
1.429
1.416

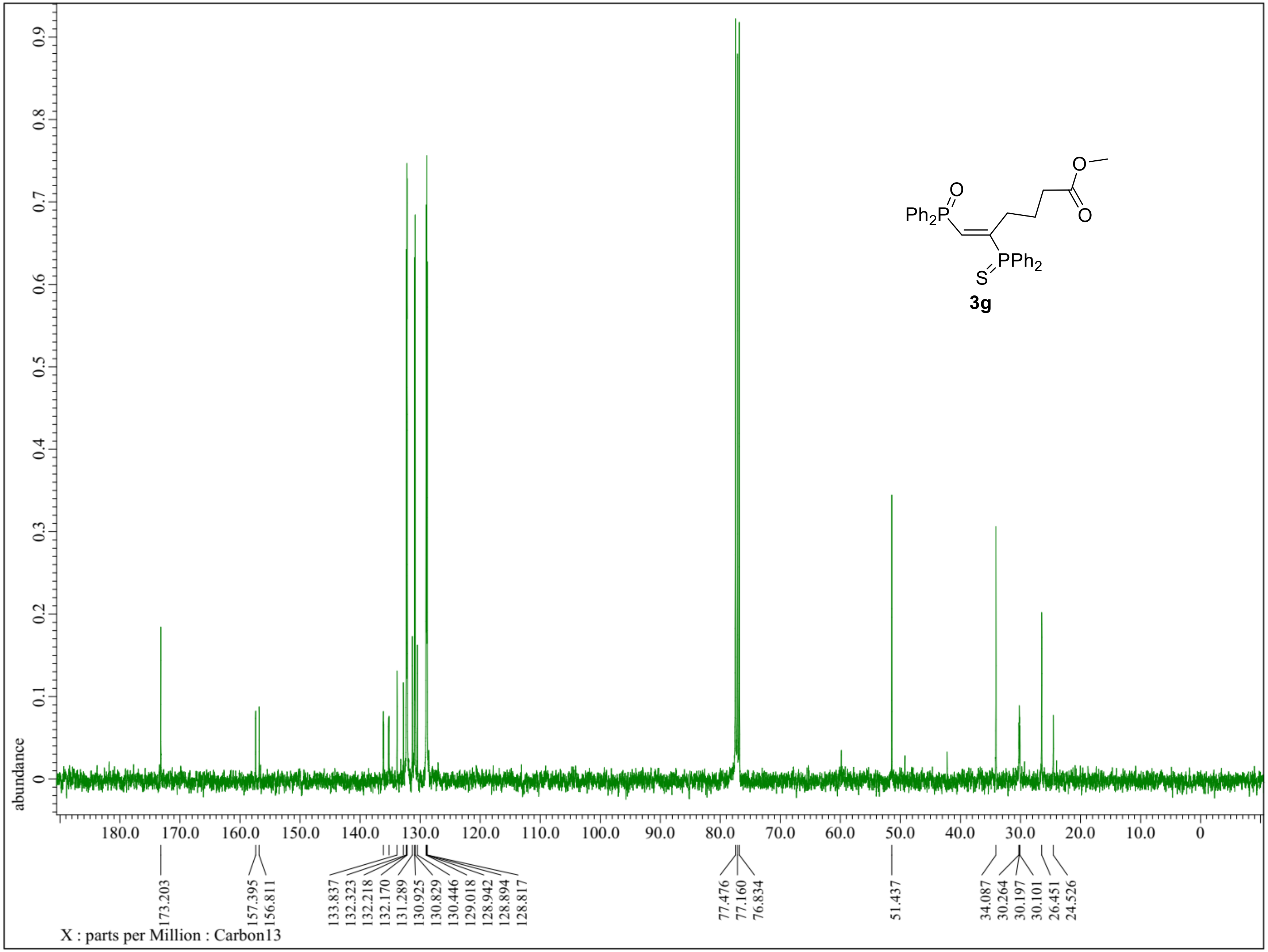
Ph2P
O
O
O
S
PPh2
3g
abundance
0.9
0.8
0.7
0.6
0.5
0.4
0.3
0.2
0.1
0
180.0 170.0 160.0 150.0 140.0 130.0 120.0 110.0 100.0 90.0 80.0 70.0 60.0 50.0 40.0 30.0 20.0 10.0 0
173.203
157.395
156.811
133.837
132.323
132.218
132.170
131.289
130.925
130.829
130.446
129.018
128.942
128.894
128.817
77.476
77.160
76.834
51.437
34.087
30.264
30.197
30.101
26.451
24.526
X : parts per Million : Carbon13

"D 0814 C5COOCh3 27 35" 1 1 D:\nmrdata\OGAWA

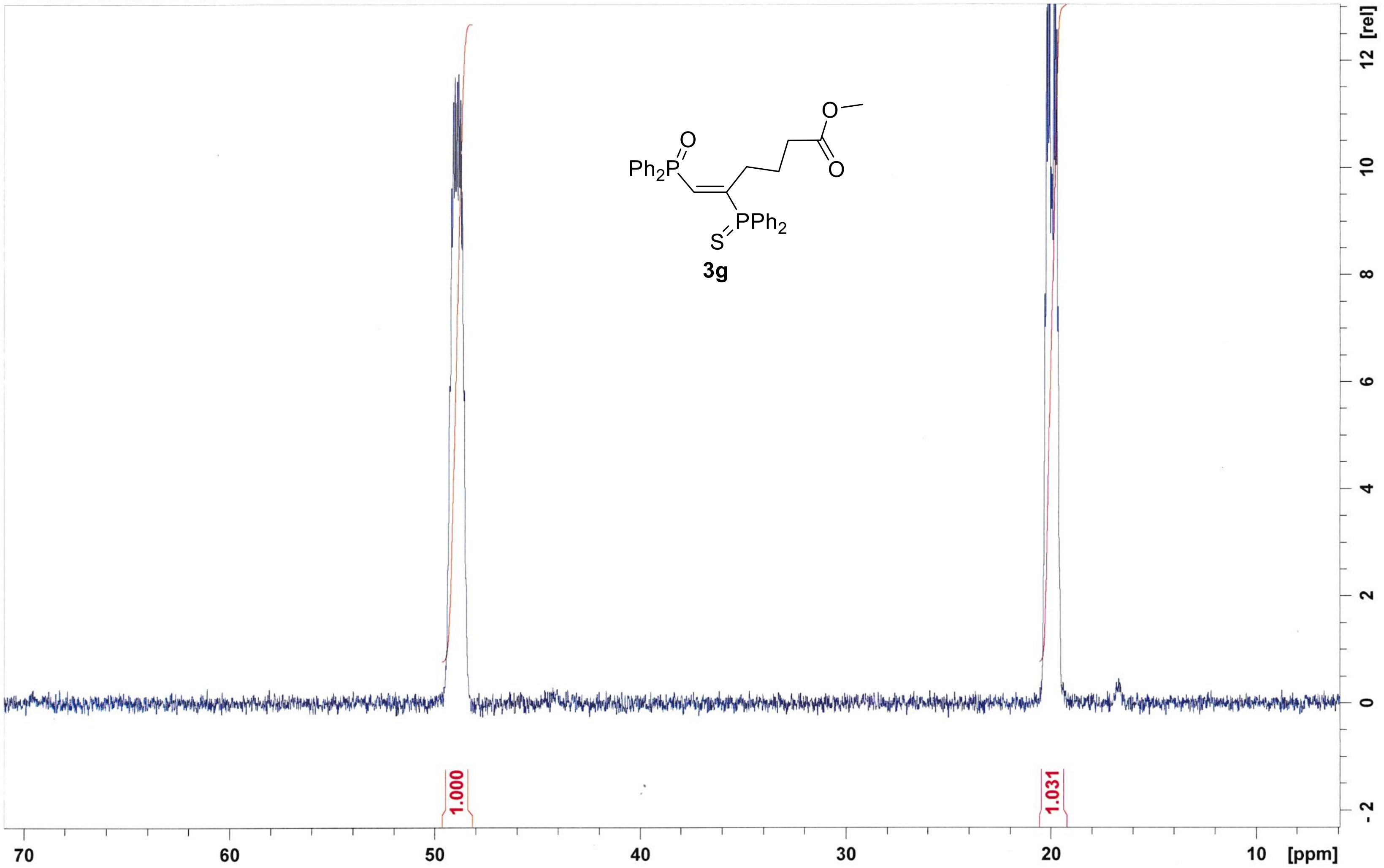

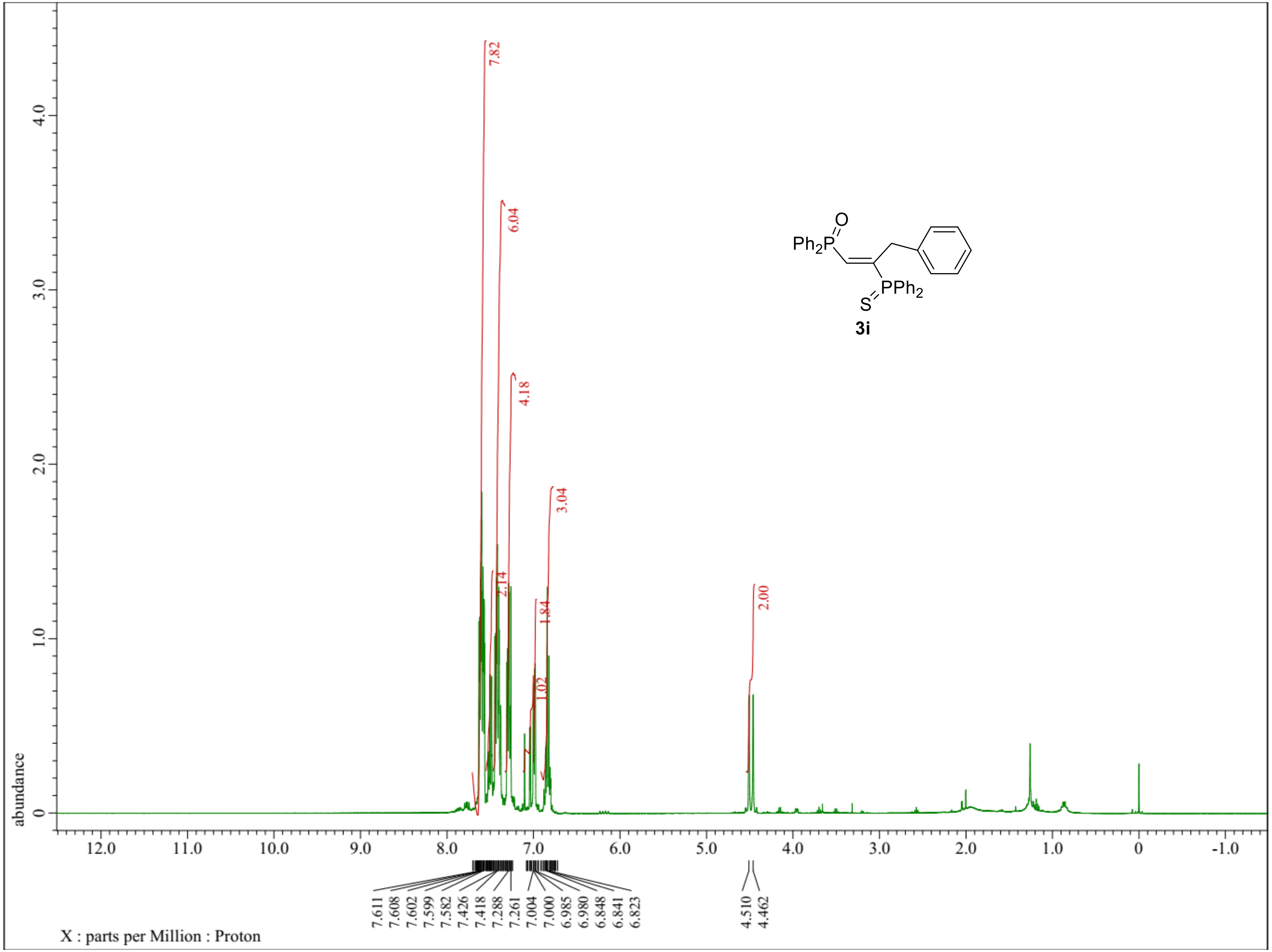

abundance
X : parts per Million : Proton
Ph2P
O
S
PPh2
3i

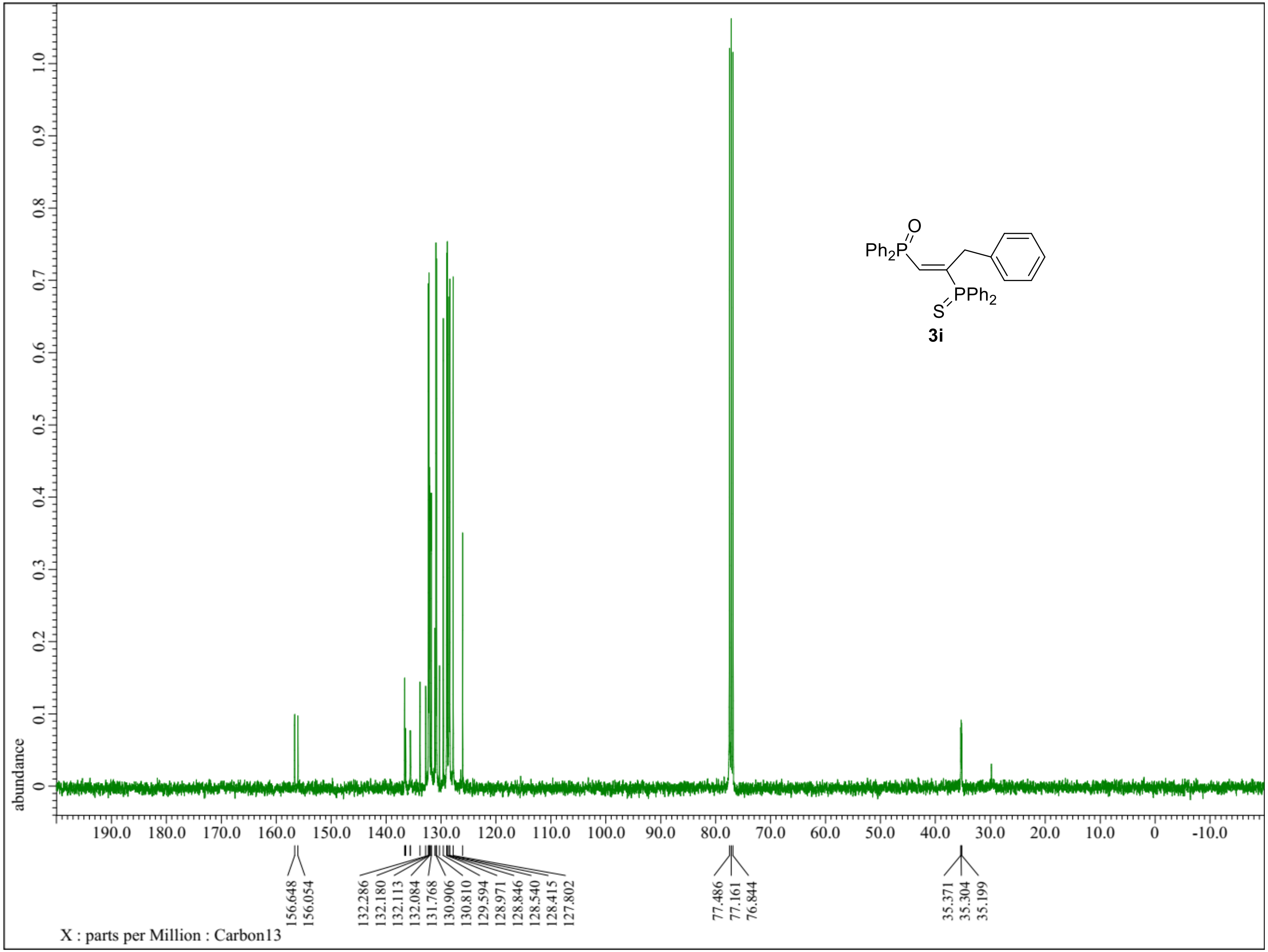

3i
abundance
X : parts per Million : Carbon13
156.648
156.054
132.286
132.180
132.113
132.084
131.768
130.906
130.810
129.594
128.971
128.846
128.540
128.415
127.802
77.486
77.161
76.844
35.371
35.304
35.199

"D 1009 PhC3 CC11" 1 1 D:\nmrdata\OGAWA\Dat

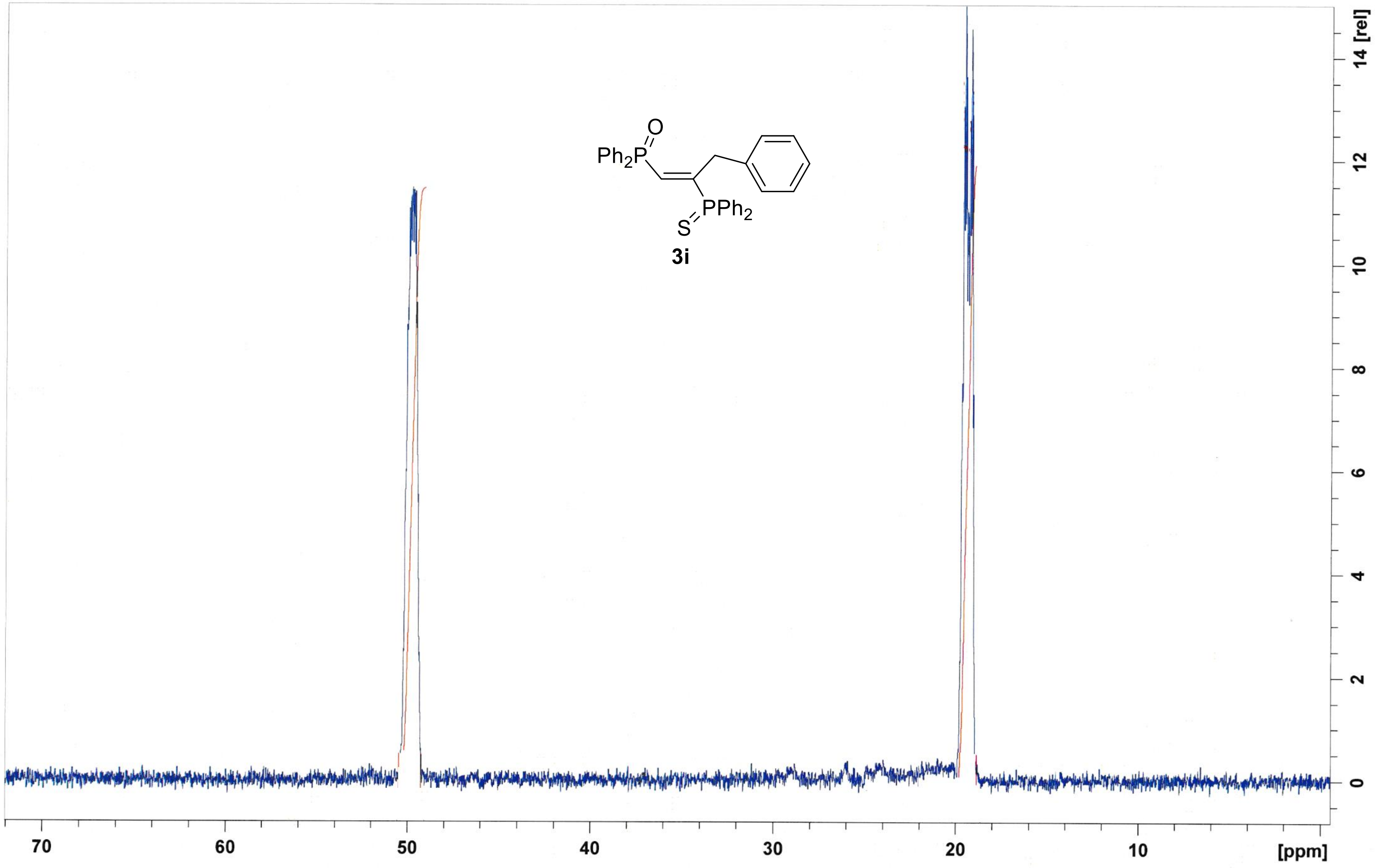

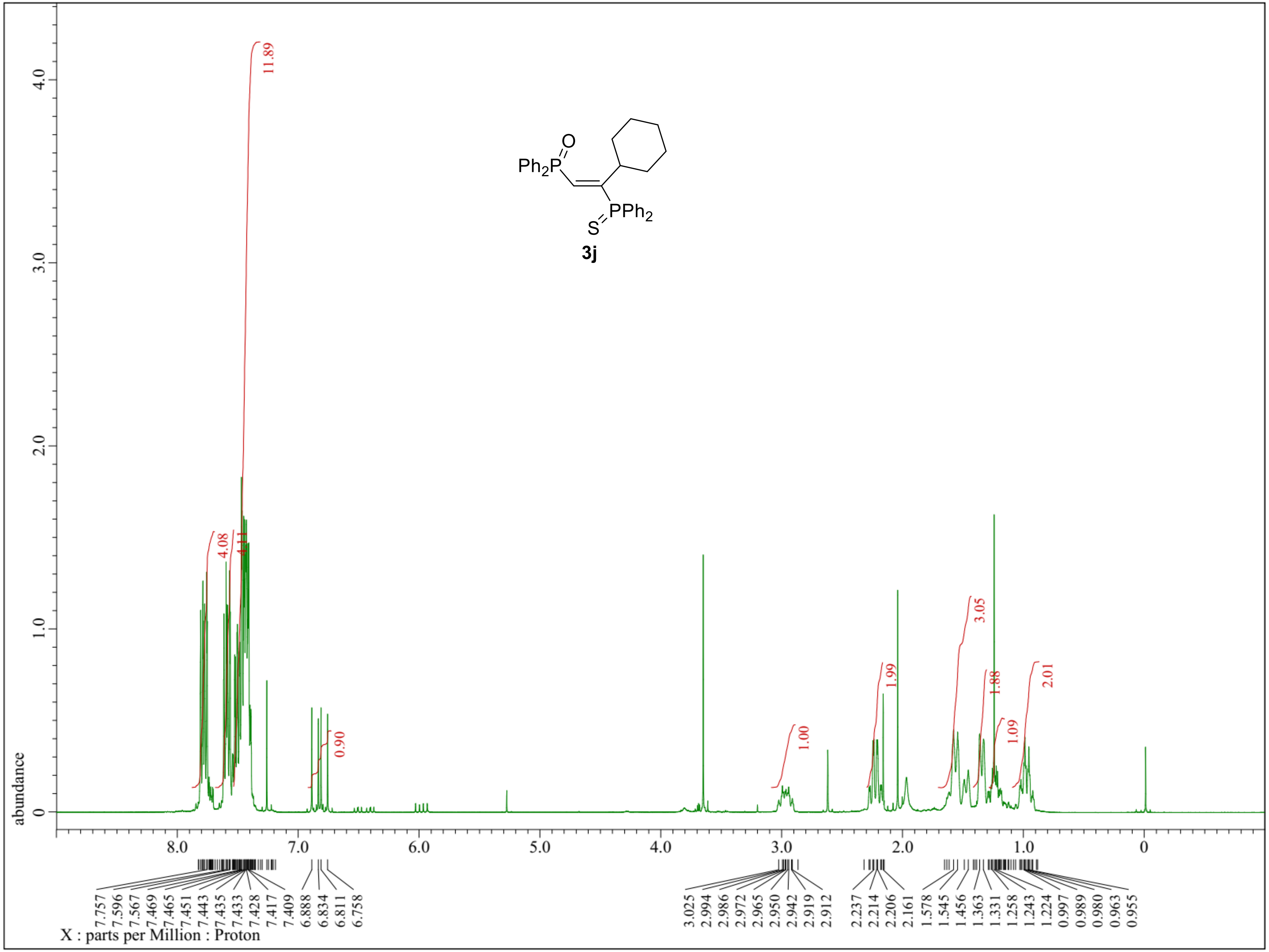

Ph2P=O
S=PPh2
3j
abundance
X : parts per Million : Proton
11.89
4.08
4.11
0.90
1.00
1.99
3.05
1.88
1.09
2.01
7.757
7.596
7.567
7.469
7.465
7.451
7.443
7.435
7.433
7.428
7.417
7.409
6.888
6.834
6.811
6.758
3.025
2.994
2.986
2.972
2.965
2.950
2.942
2.919
2.912
2.237
2.214
2.206
2.161
1.578
1.545
1.456
1.363
1.331
1.258
1.243
1.224
0.997
0.989
0.980
0.963
0.955

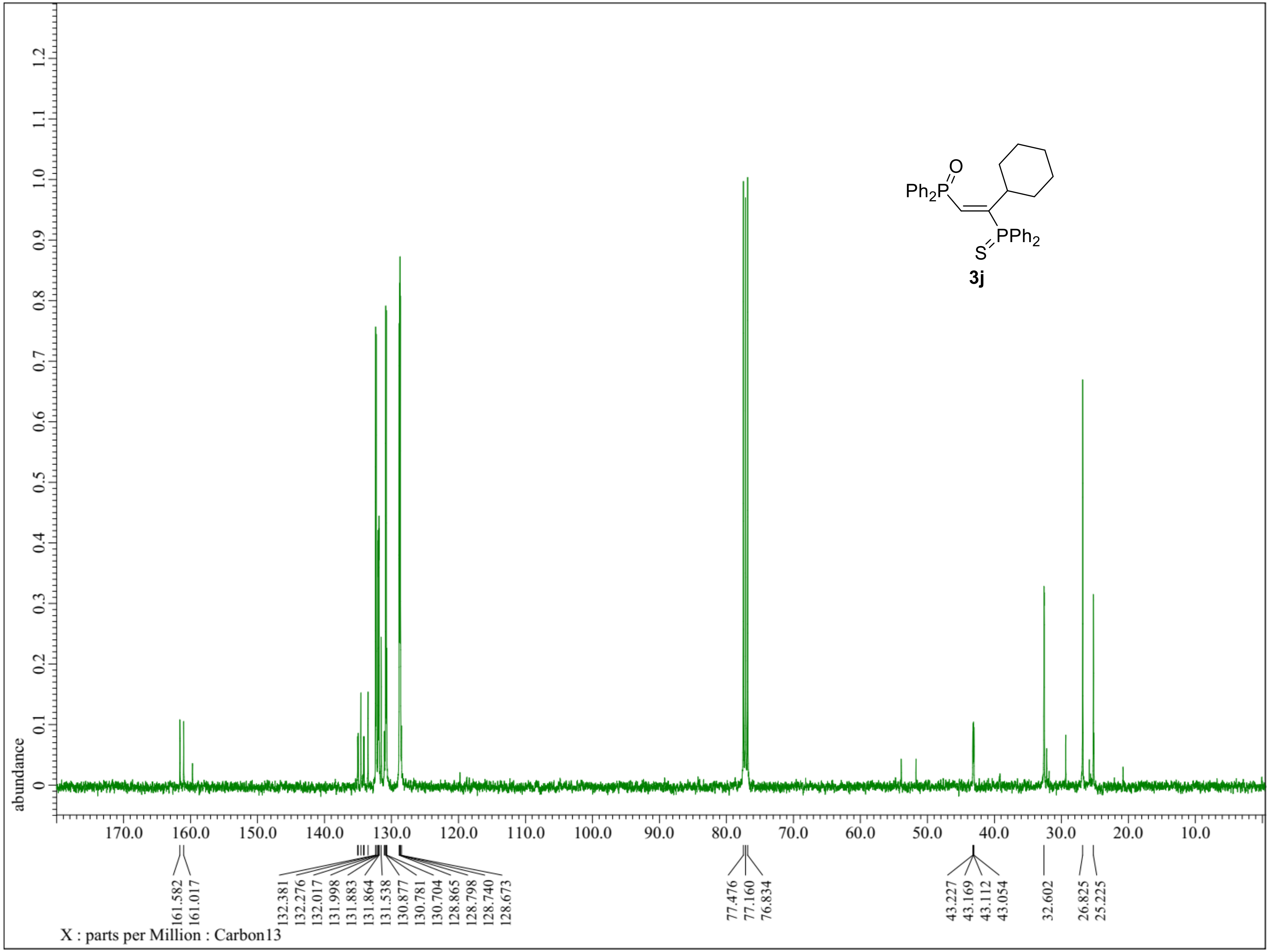

abundance
0
0.1
0.2
0.3
0.4
0.5
0.6
0.7
0.8
0.9
1.0
1.1
1.2
Ph2P O
PPh2 S
3j
170.0
160.0
150.0
140.0
130.0
120.0
110.0
100.0
90.0
80.0
70.0
60.0
50.0
40.0
30.0
20.0
10.0
161.582
161.017
132.381
132.276
132.017
131.998
131.883
131.864
131.538
130.877
130.781
130.704
128.865
128.798
128.740
128.673
77.476
77.160
76.834
43.227
43.169
43.112
43.054
32.602
26.825
25.225
X : parts per Million : Carbon13

"D 0903 cyclohex" 1 1 D:\nmrdata\OGAWA

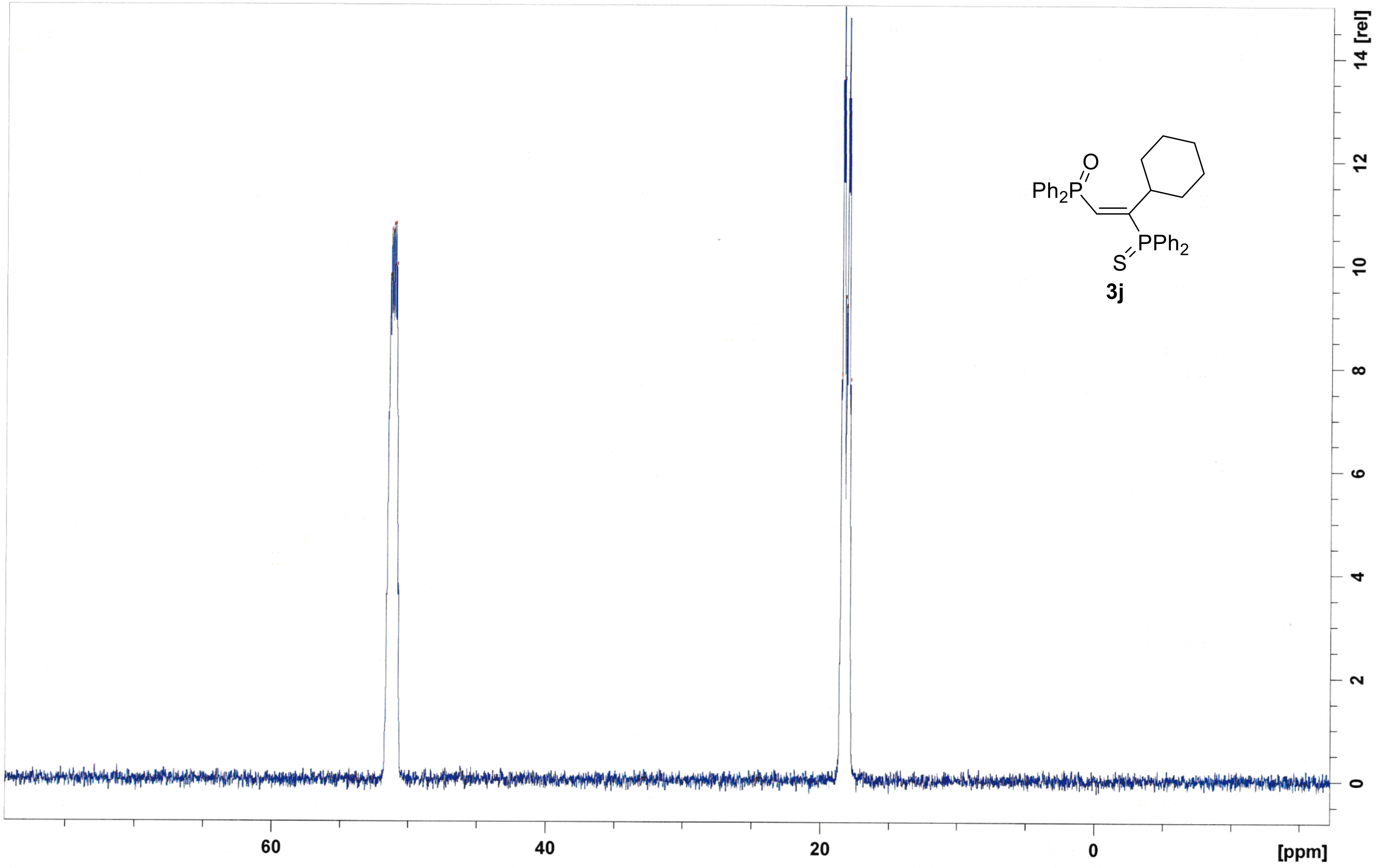

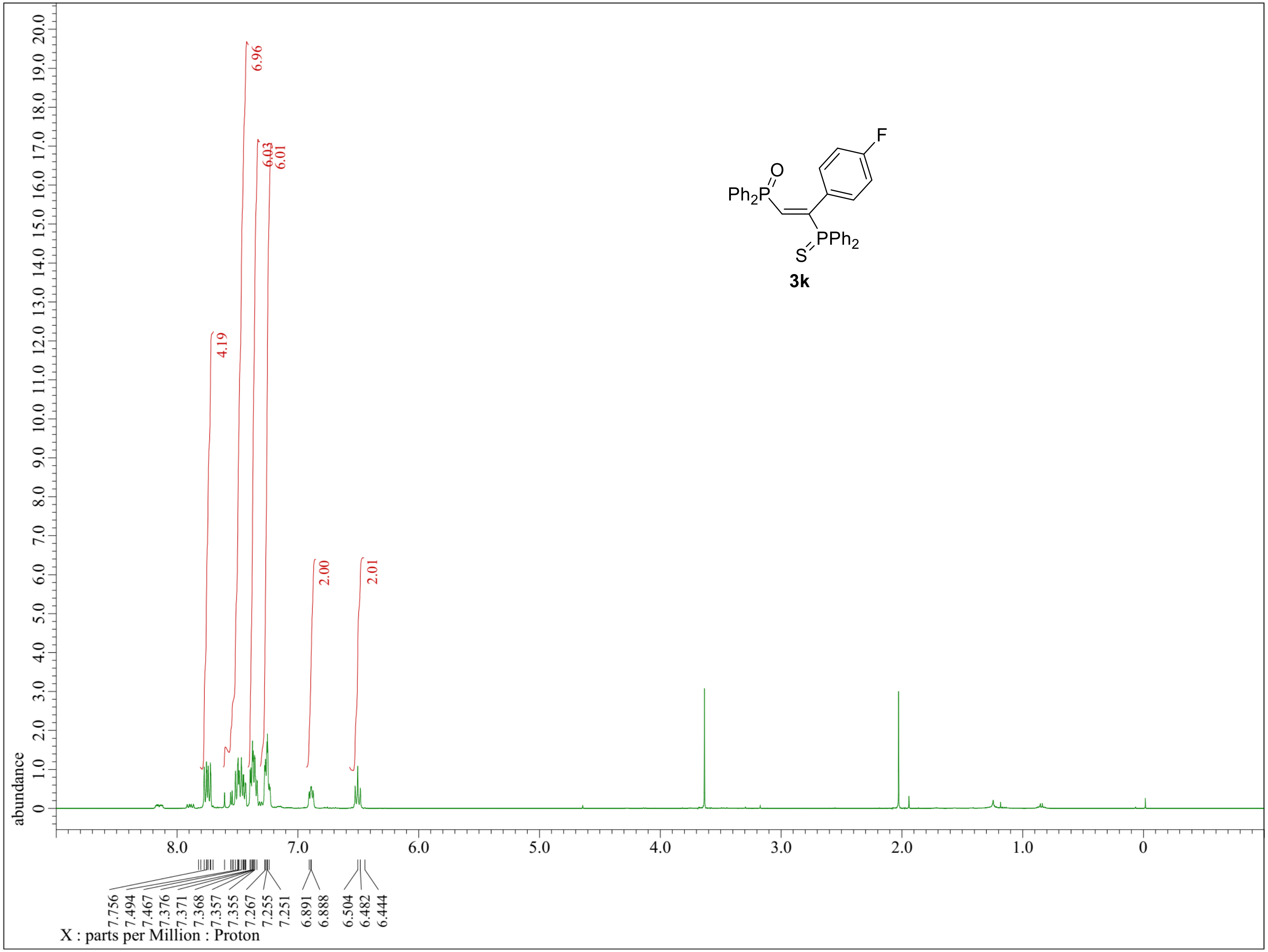

6.96
6.03
6.01
4.19
2.00
2.01
Ph2P
O
F
S
PPh2
3k
abundance
8.0
7.0
6.0
5.0
4.0
3.0
2.0
1.0
0
7.756
7.494
7.467
7.376
7.371
7.368
7.357
7.355
7.267
7.255
7.251
6.891
6.888
6.504
6.482
6.444
X : parts per Million : Proton

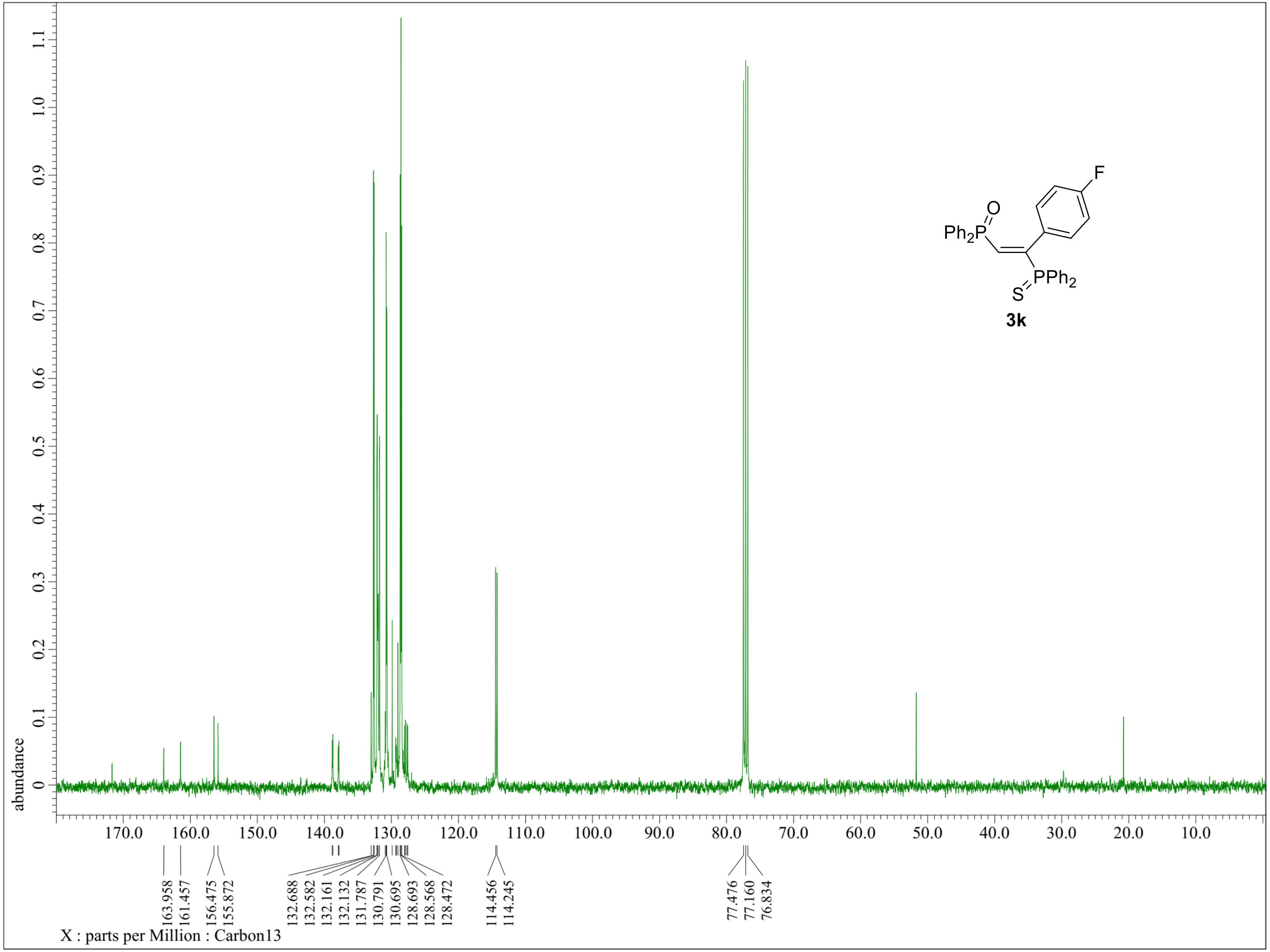

Ph2P
O
F
S
PPh2
3k
abundance
0
0.1
0.2
0.3
0.4
0.5
0.6
0.7
0.8
0.9
1.0
1.1
170.0
160.0
150.0
140.0
130.0
120.0
110.0
100.0
90.0
80.0
70.0
60.0
50.0
40.0
30.0
20.0
10.0
163.958
161.457
156.475
155.872
132.688
132.582
132.161
132.132
131.787
130.791
130.695
128.693
128.568
128.472
114.456
114.245
77.476
77.160
76.834
X : parts per Million : Carbon13

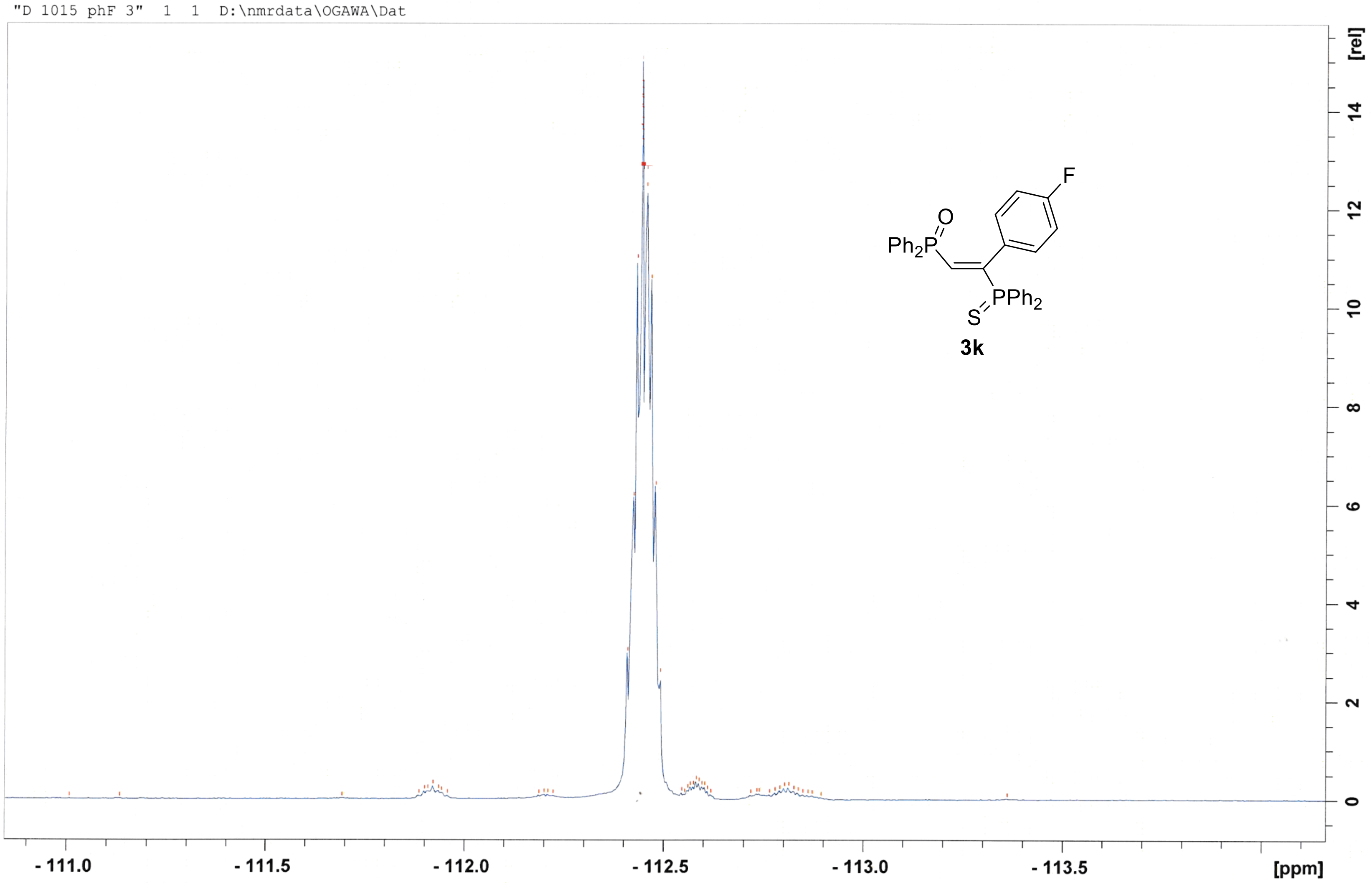
"D 1015 phF 3" 1 1 D:\nmrdata\OGAWA\Dat
[rel]
14
12
10
8
6
4
2
0
- 111.0
- 111.5
- 112.0
- 112.5
- 113.0
- 113.5
[ppm]
F
O
Ph2P
PPh2
S
3k

"D 10vv PhF 1820" 1 1 D:\nmrdata\OGAWA\Dat

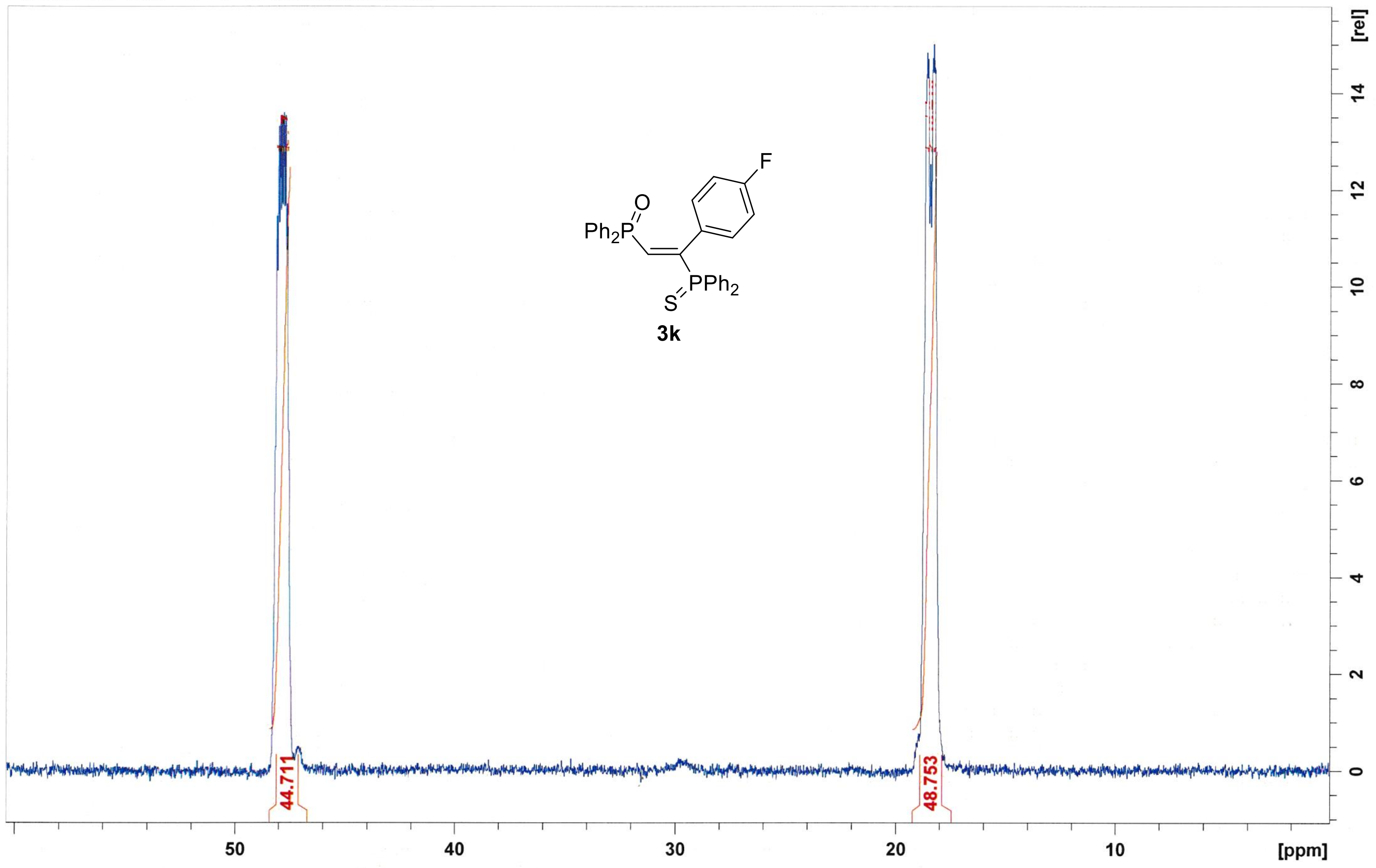

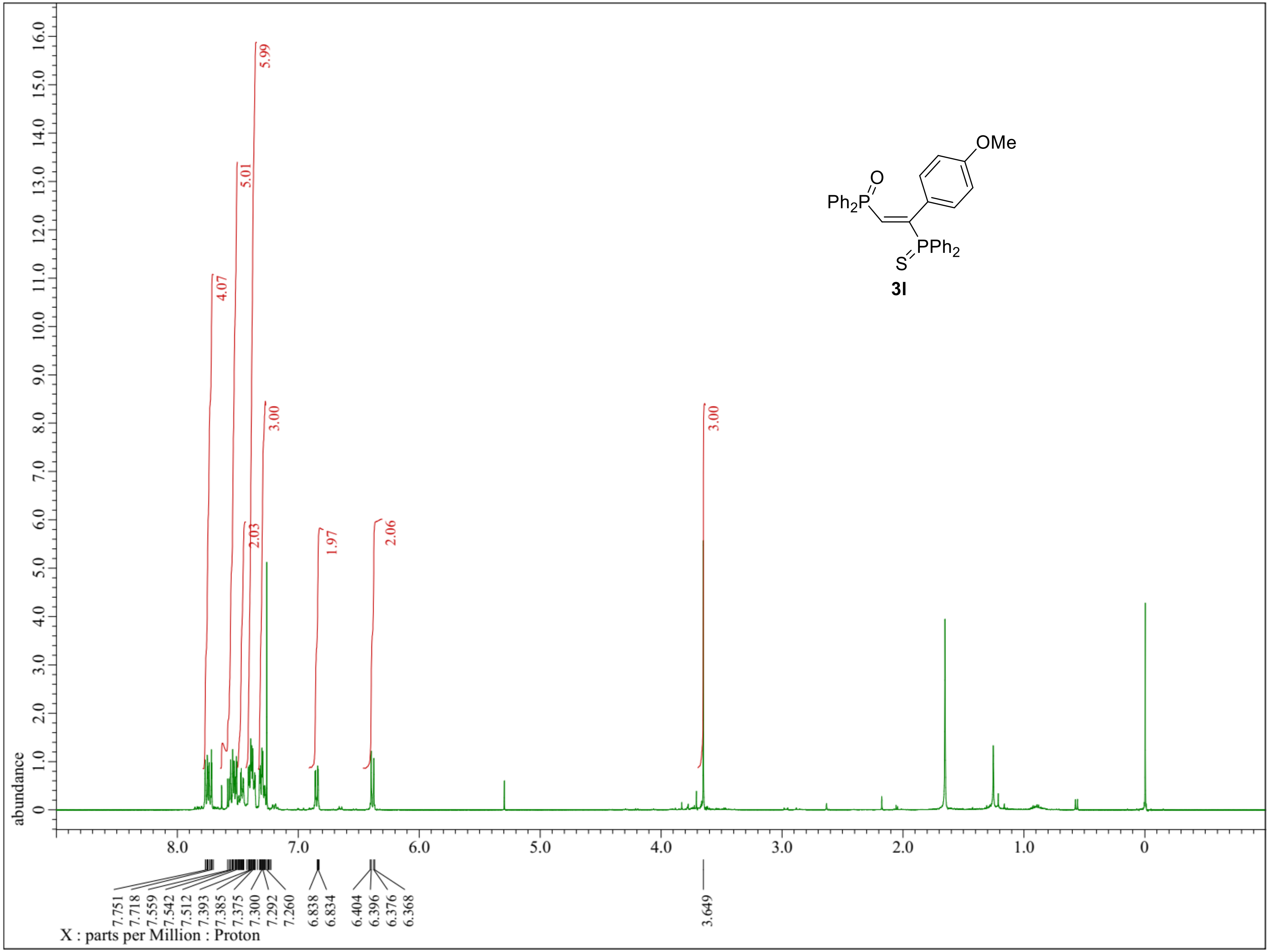
OMe
Ph2P
O
PPh2
S
3l
abundance
X : parts per Million : Proton
7.751
7.718
7.559
7.542
7.512
7.393
7.385
7.375
7.300
7.292
7.260
6.838
6.834
6.404
6.396
6.376
6.368
3.649
4.07
5.01
5.99
2.03
3.00
1.97
2.06
3.00

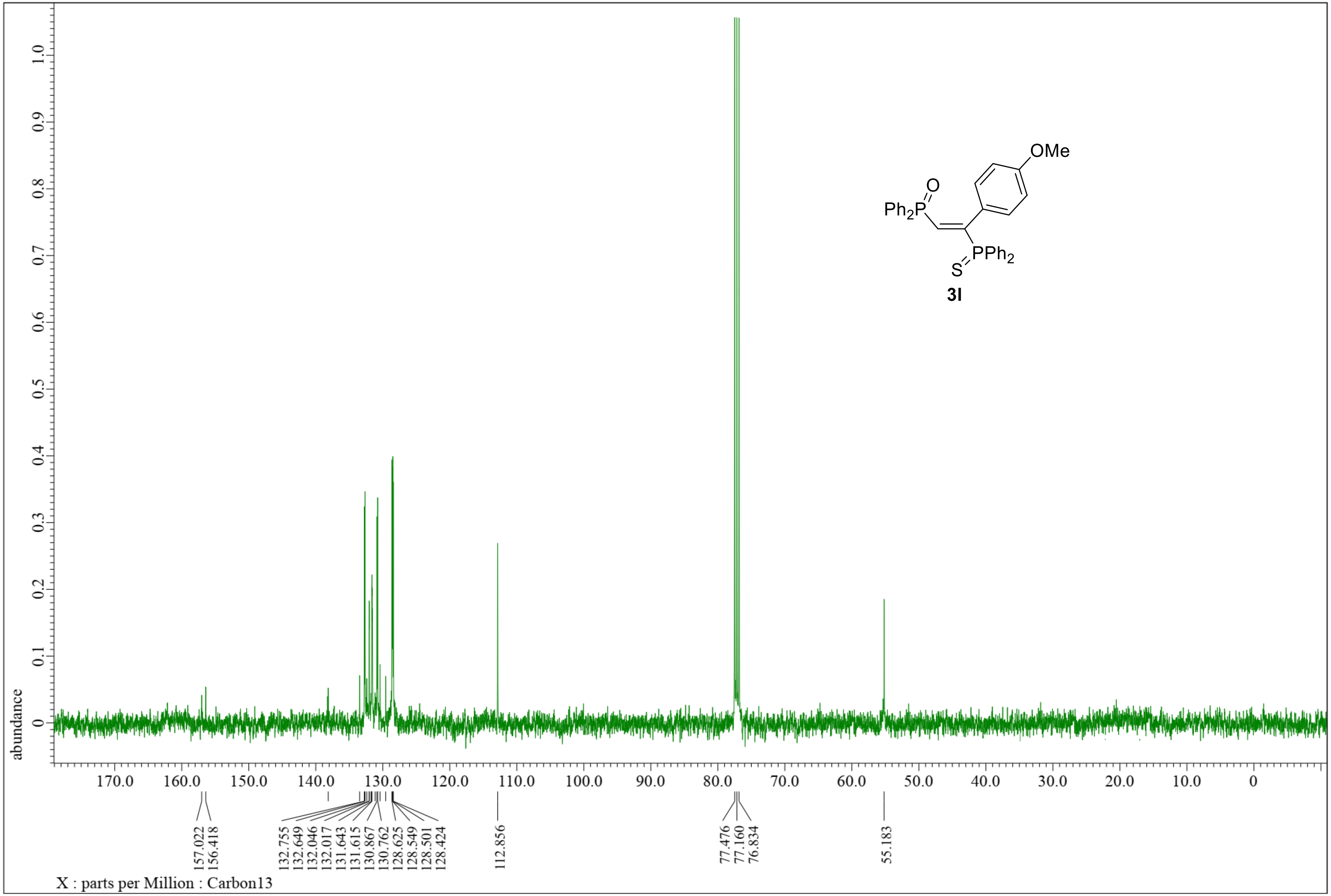

OMe
Ph2P
O
PPh2
S
3l
abundance
X : parts per Million : Carbon13
157.022
156.418
132.755
132.649
132.046
132.017
131.643
131.615
130.867
130.762
128.625
128.549
128.501
128.424
112.856
77.476
77.160
76.834
55.183

"D 0811 MeO 1" 1 1 D:\nmrdata\OGAWA

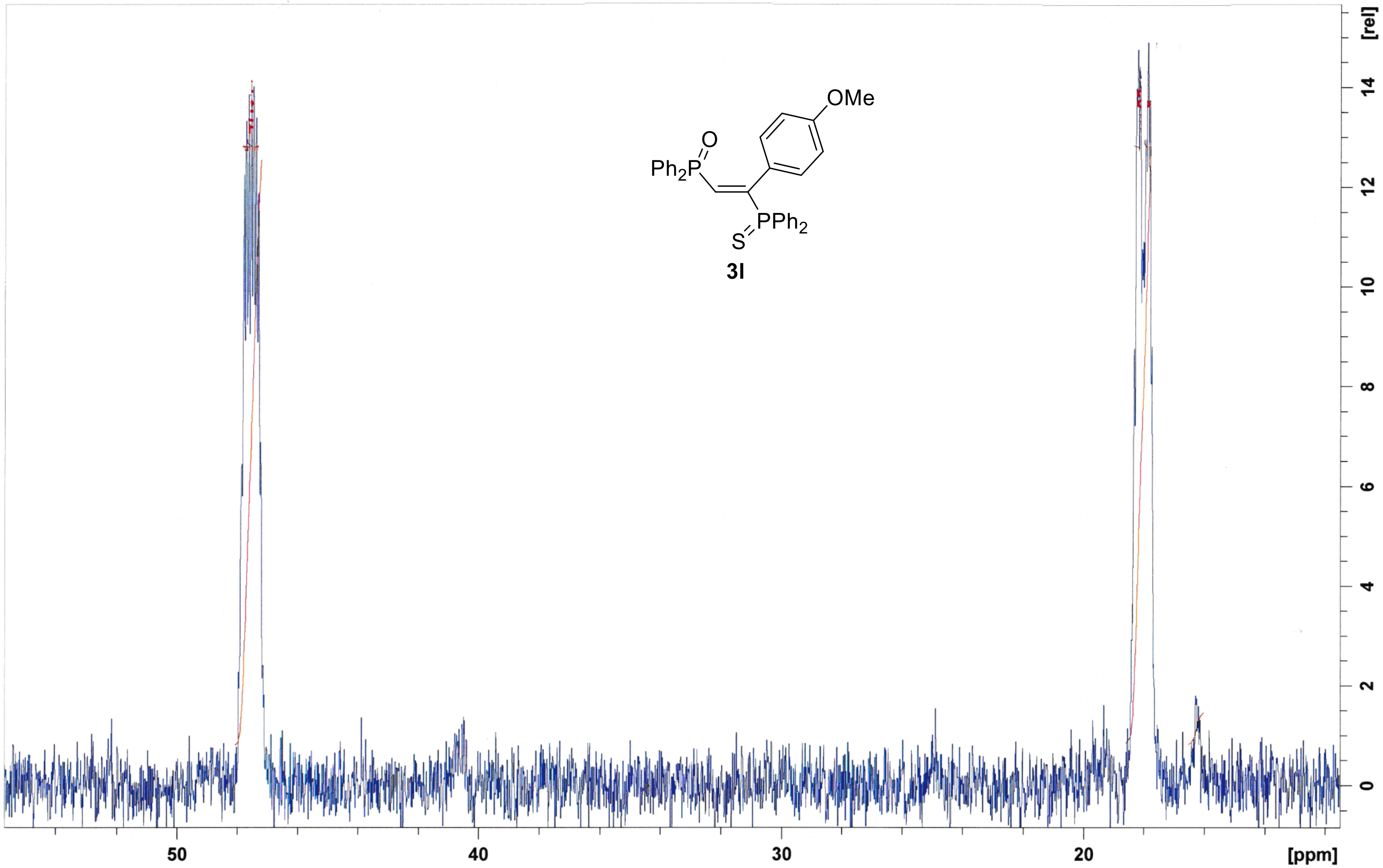

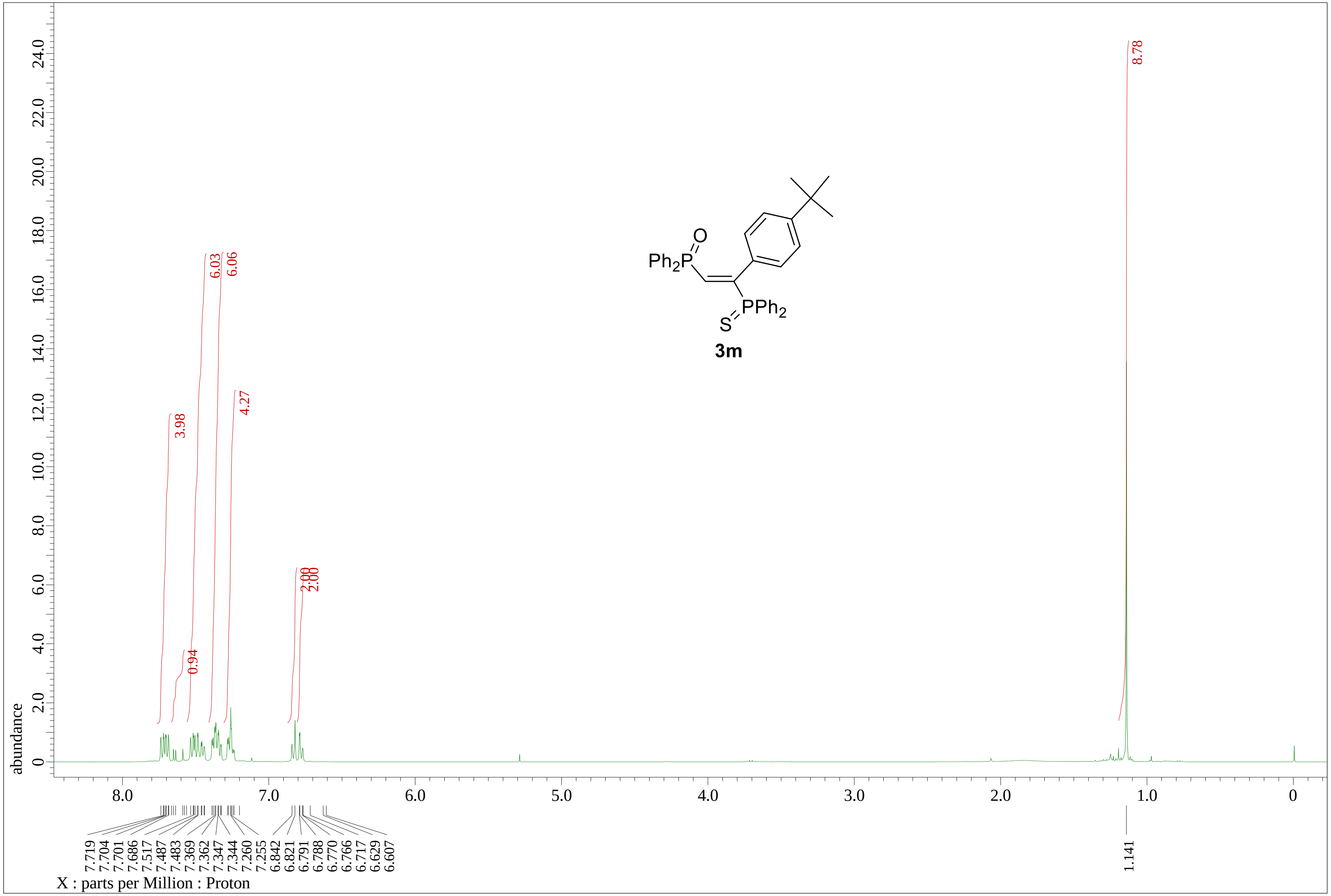

3m
abundance
X : parts per Million : Proton
8.78
6.03
6.06
3.98
4.27
0.94
2.00
2.00
7.719
7.704
7.701
7.686
7.517
7.487
7.483
7.369
7.362
7.347
7.344
7.260
7.255
6.842
6.821
6.791
6.788
6.770
6.766
6.717
6.629
6.607
1.141

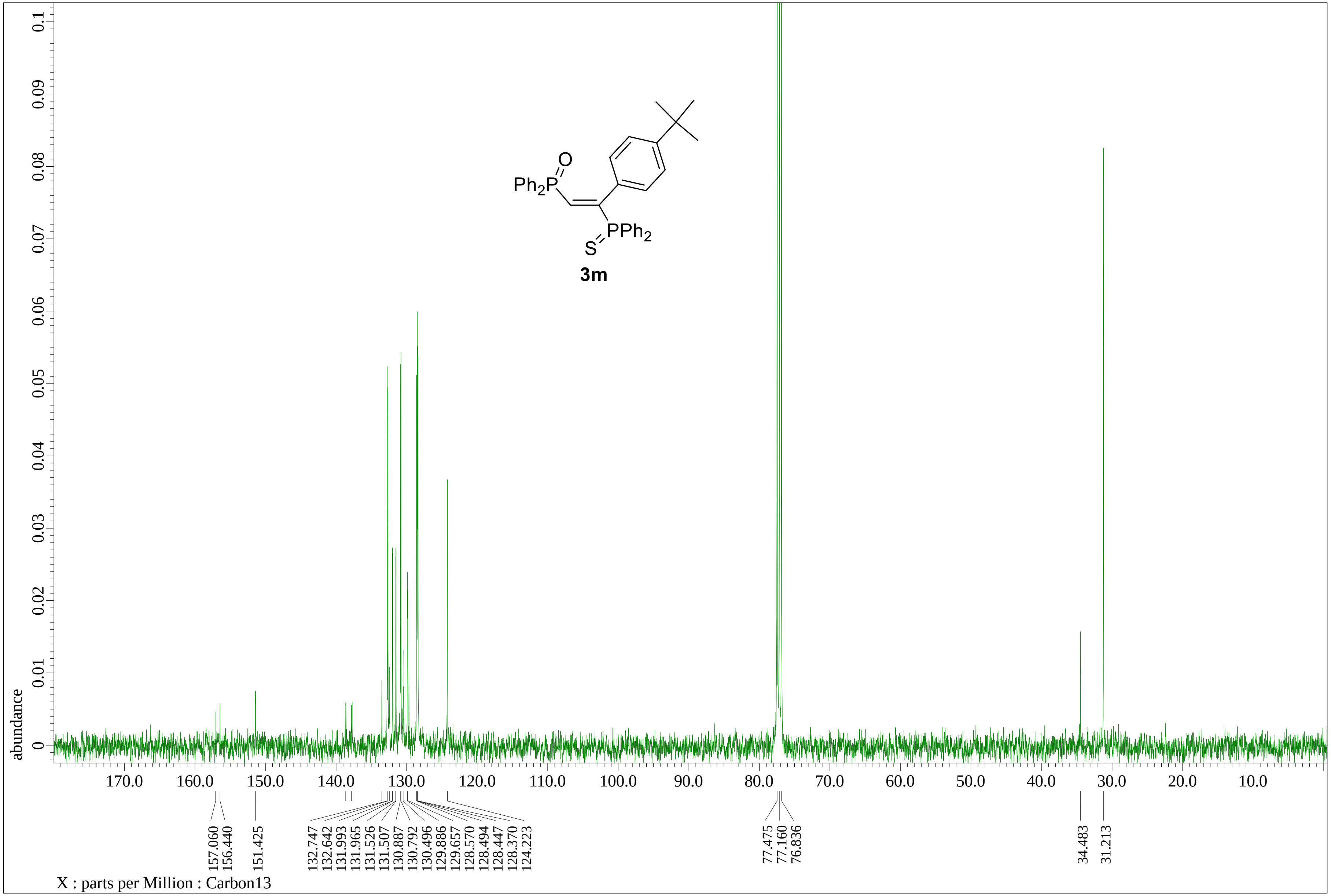
Ph2P=O
S=PPh2
3m
abundance
0
0.01
0.02
0.03
0.04
0.05
0.06
0.07
0.08
0.09
0.1
170.0
160.0
150.0
140.0
130.0
120.0
110.0
100.0
90.0
80.0
70.0
60.0
50.0
40.0
30.0
20.0
10.0
157.060
156.440
151.425
132.747
132.642
131.993
131.965
131.526
131.507
130.887
130.792
130.496
129.886
129.657
128.570
128.494
128.447
128.370
124.223
77.475
77.160
76.836
34.483
31.213
X : parts per Million : Carbon13

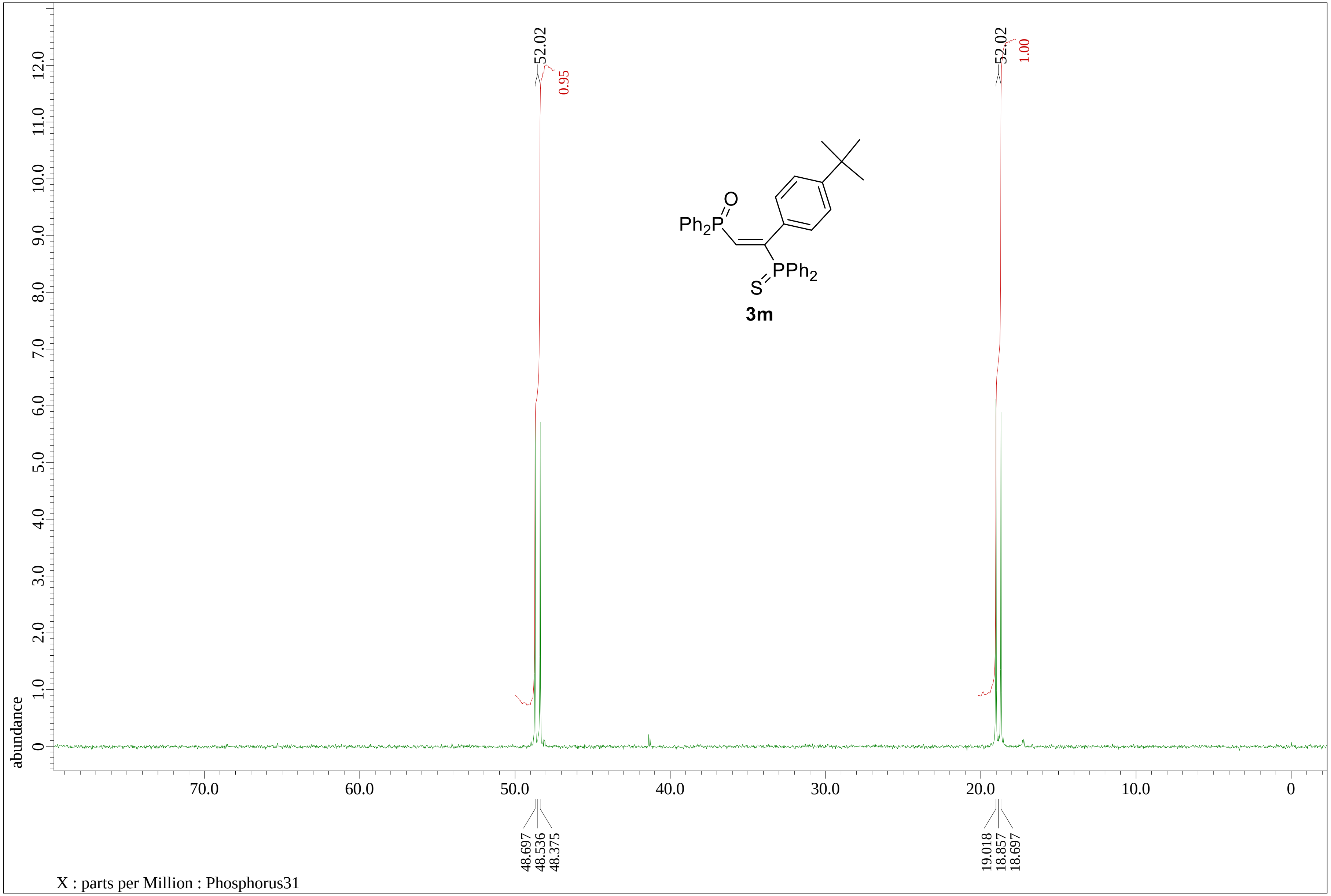
52.02
0.95
52.02
1.00
Ph2P
O
PPh2
S
3m
abundance
12.0
11.0
10.0
9.0
8.0
7.0
6.0
5.0
4.0
3.0
2.0
1.0
0
70.0
60.0
50.0
40.0
30.0
20.0
10.0
0
48.697
48.536
48.375
19.018
18.857
18.697
X : parts per Million : Phosphorus31

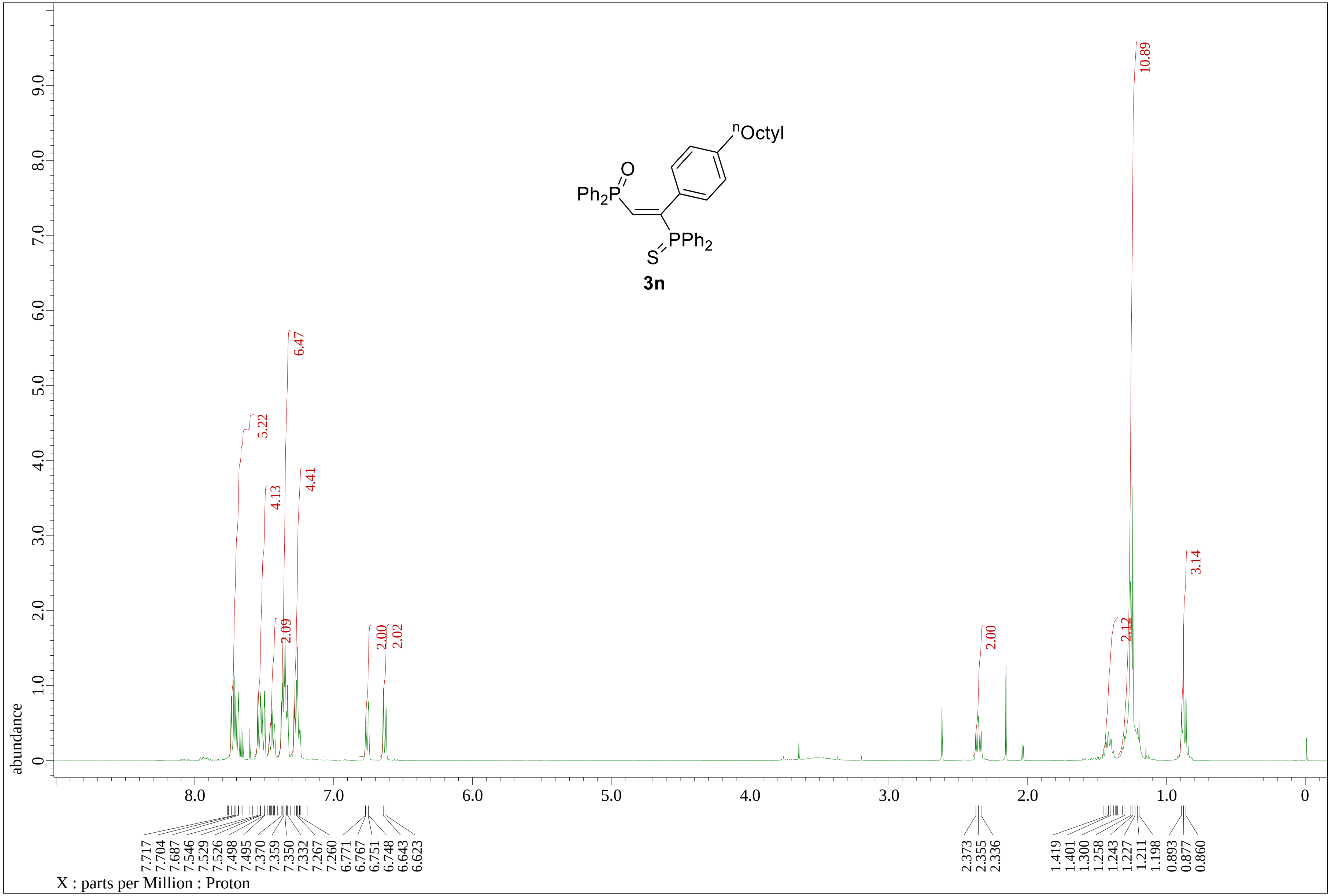

ⁿOctyl
O
Ph2P
PPh2
S
3n
abundance
X : parts per Million : Proton
10.89
6.47
5.22
4.13
4.41
2.09
2.00
2.02
2.00
2.12
3.14
7.717
7.704
7.687
7.546
7.529
7.526
7.498
7.495
7.370
7.359
7.350
7.332
7.267
7.260
6.771
6.767
6.751
6.748
6.643
6.623
2.373
2.355
2.336
1.419
1.401
1.300
1.258
1.243
1.227
1.211
1.198
0.893
0.877
0.860

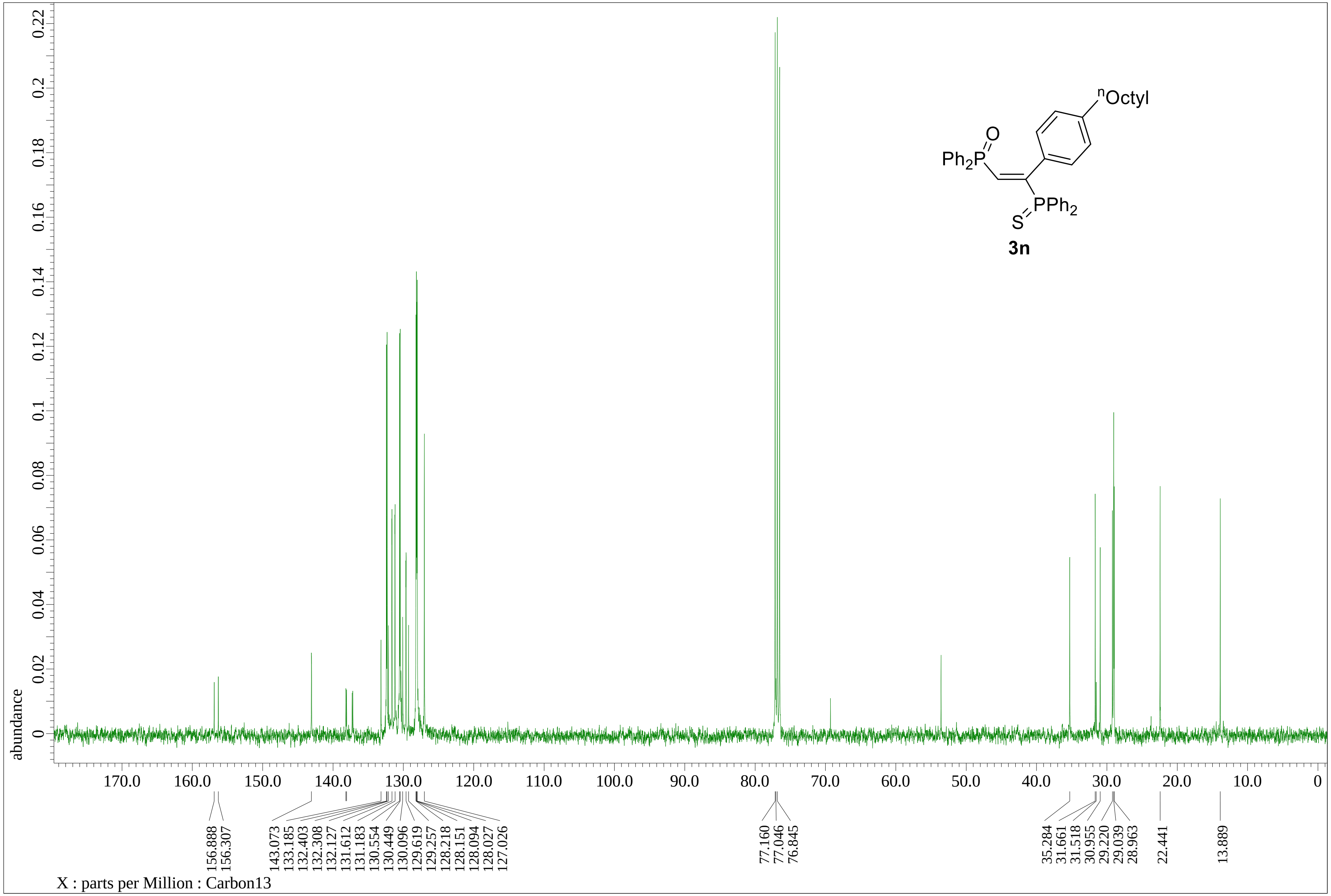

abundance
X : parts per Million : Carbon13
3n

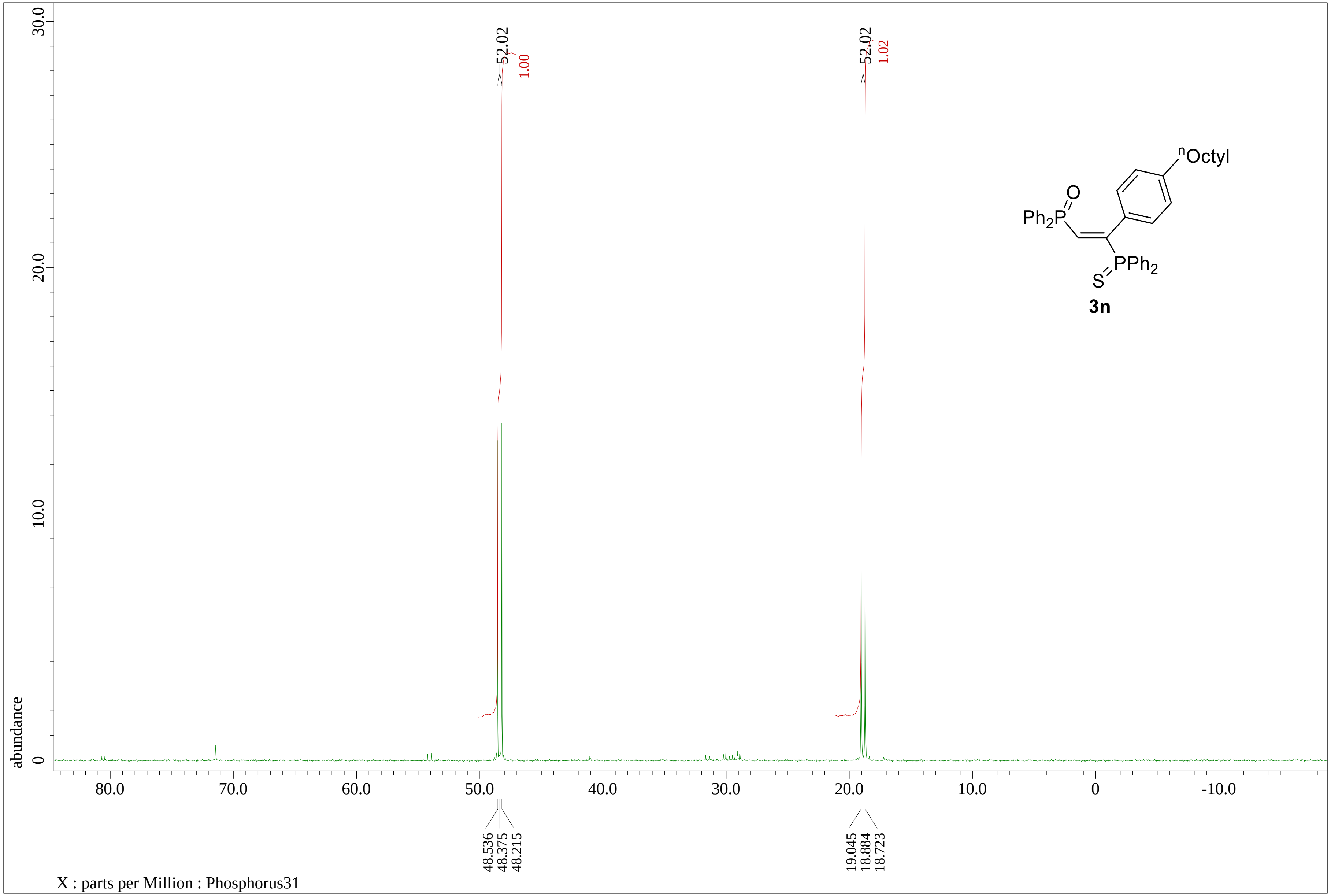

52.02
1.00
52.02
1.02
Ph2P
O
nOctyl
S
PPh2
3n
30.0
20.0
10.0
0
abundance
80.0
70.0
60.0
50.0
40.0
30.0
20.0
10.0
0
-10.0
48.536
48.375
48.215
19.045
18.884
18.723
X : parts per Million : Phosphorus31

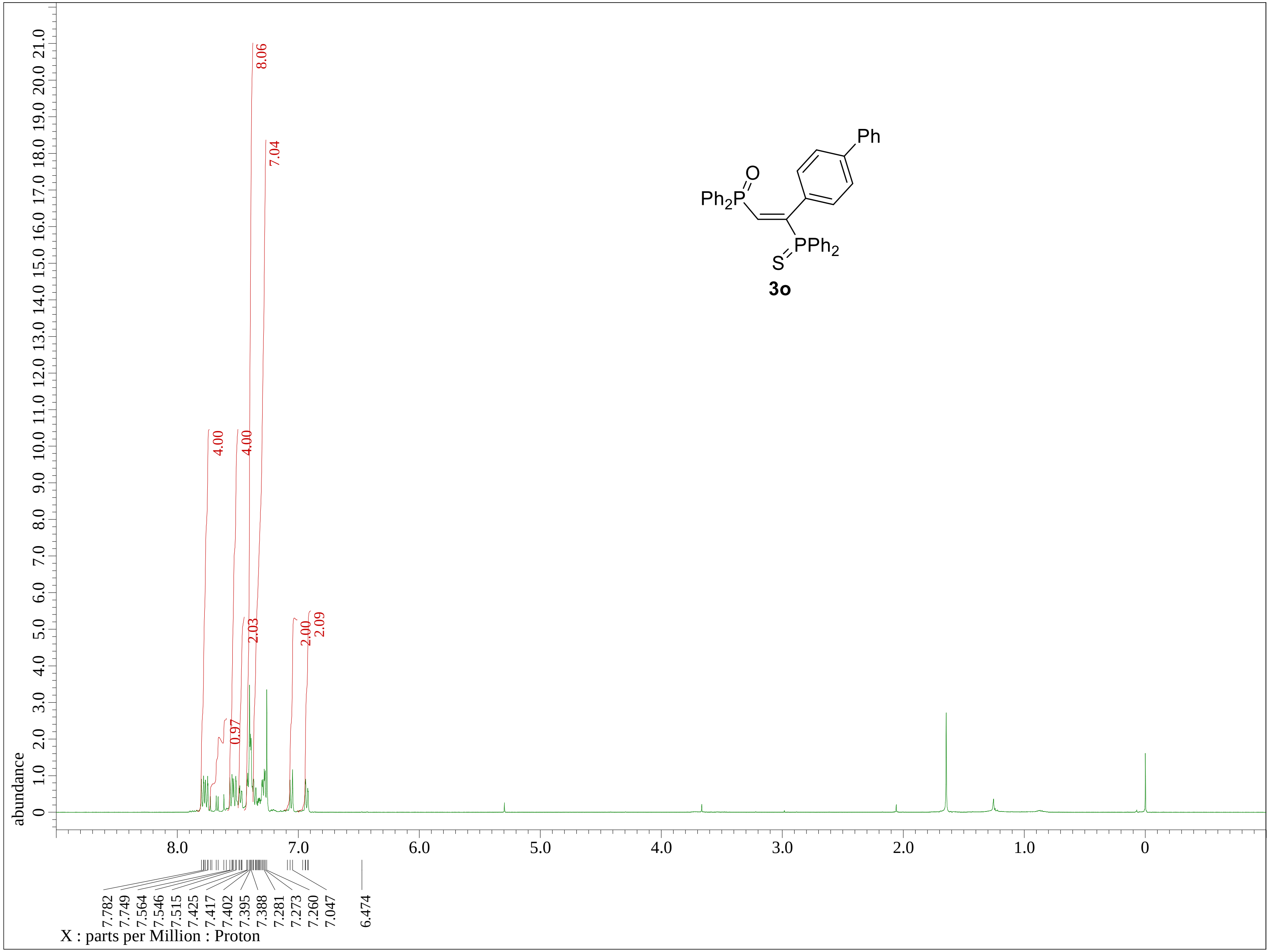

Ph
O
Ph2P
PPh2
S
3o
abundance
X : parts per Million : Proton
4.00
4.00
8.06
7.04
0.97
2.03
2.00
2.09
7.782
7.749
7.564
7.546
7.515
7.425
7.417
7.402
7.395
7.388
7.281
7.273
7.260
7.047
6.474

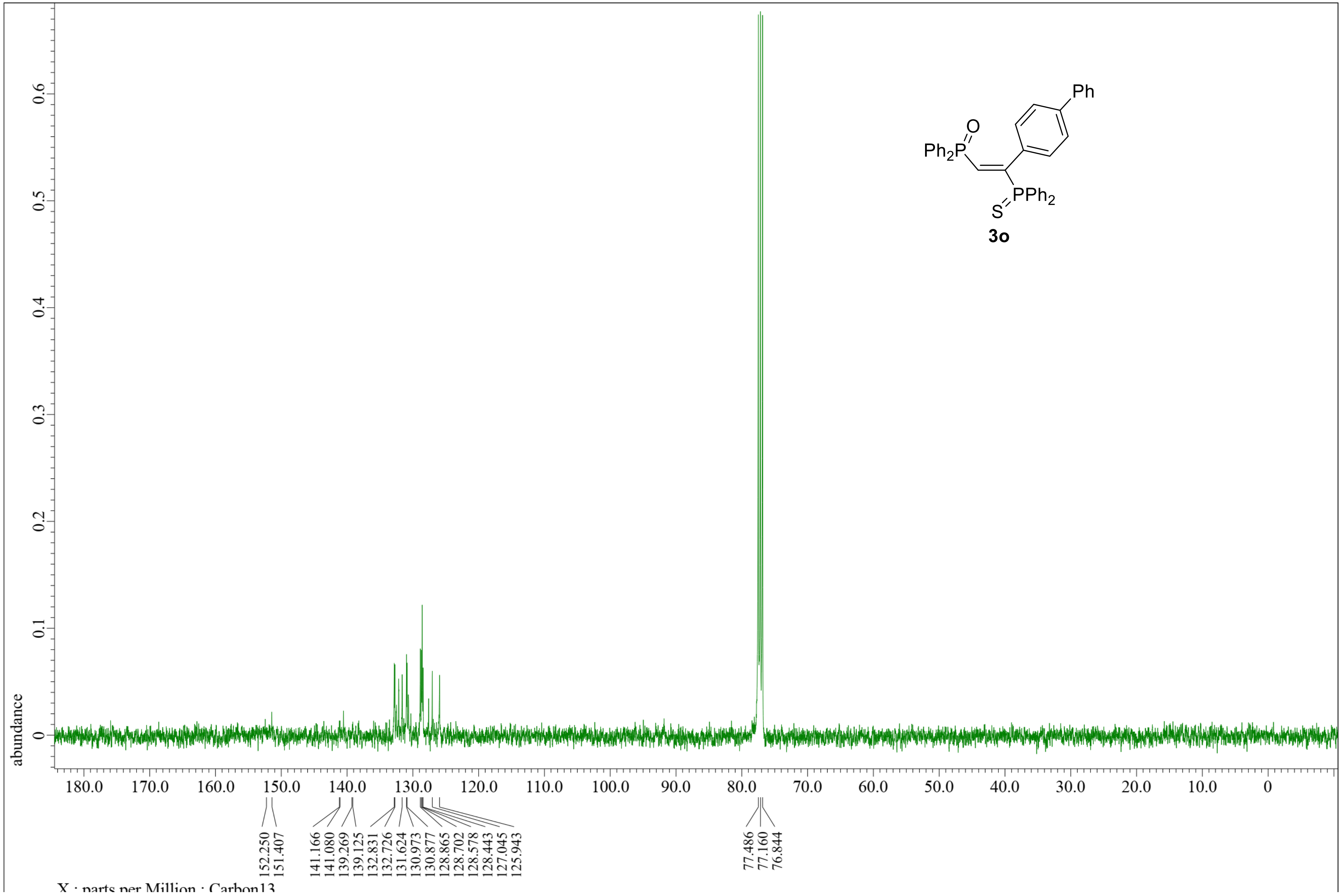
abundance
0
0.1
0.2
0.3
0.4
0.5
0.6
180.0
170.0
160.0
150.0
140.0
130.0
120.0
110.0
100.0
90.0
80.0
70.0
60.0
50.0
40.0
30.0
20.0
10.0
0
152.250
151.407
141.166
141.080
139.269
139.125
132.831
132.726
131.624
130.973
130.877
128.865
128.702
128.578
128.443
127.045
125.943
77.486
77.160
76.844
X : parts per Million : Carbon13
Ph
O
Ph2P
PPh2
S
3o

"D 1006 BiPh" 1 1 D:\nmrdata\OGAWA\Dat

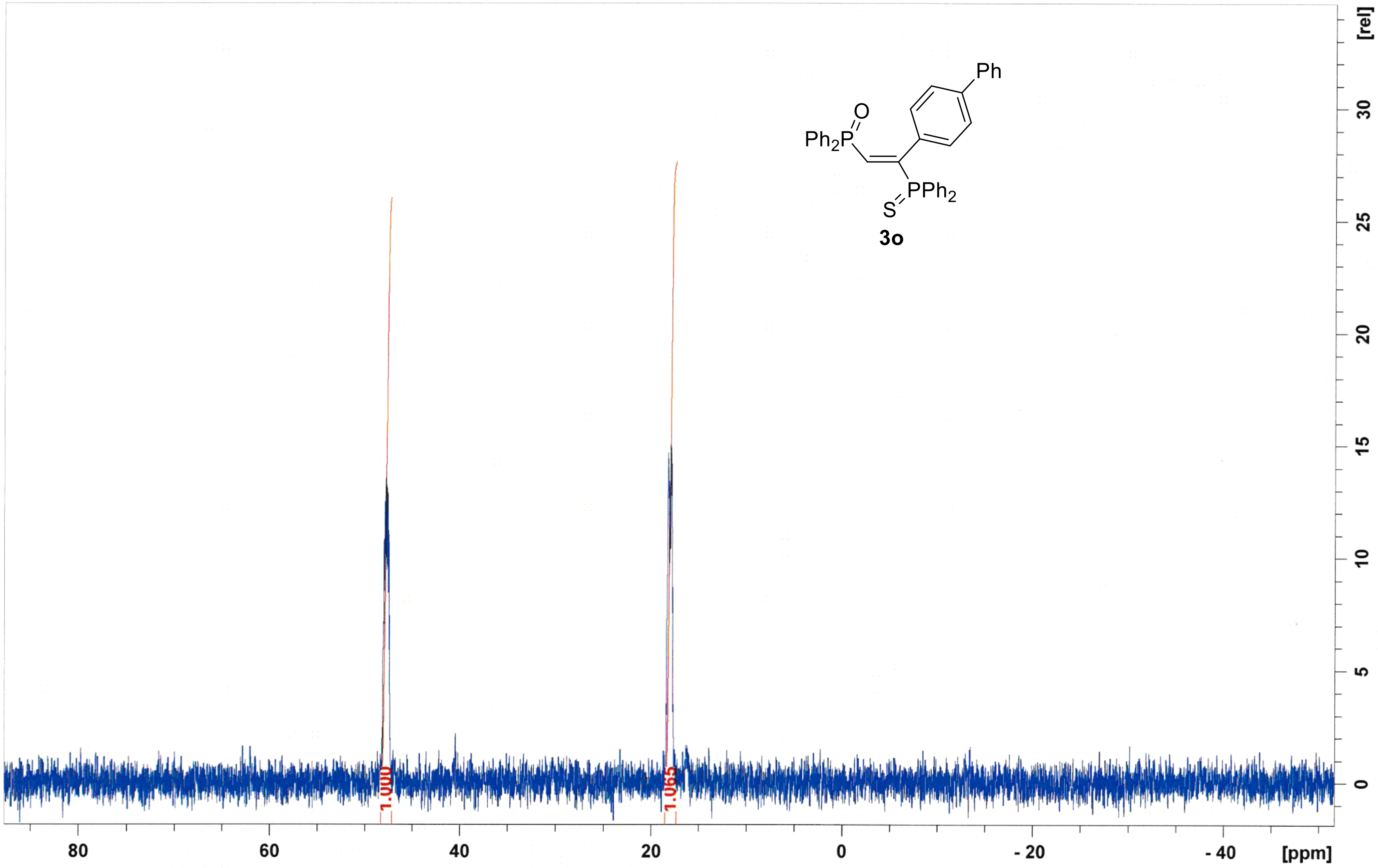

Supplement: File 1 — Characterization data and copies of NMR spectra. [file Beilstein_J_Org_Chem-17-866-s001.pdf]
